# Numerosity adaptation suppresses early visual responses

**DOI:** 10.1038/s42003-025-09041-4

**Published:** 2025-11-24

**Authors:** Liangyou Zhang, Evi Hendrikx, Yizhen Wang, Surya Gayet, Serge O. Dumoulin, Ben M. Harvey

**Affiliations:** 1 Experimental Psychology, Helmholtz Institute, Utrecht University, Utrecht, Netherlands; 2 Computational Cognitive Neuroscience and Neuroimaging, Netherlands Institute for Neuroscience, Amsterdam, Netherlands; 3 School of Psychology, South China Normal University, Guangzhou, China; 4 Spinoza Centre for Neuroimaging, Amsterdam, Netherlands; 5 Experimental and Applied Psychology, Vrije Universiteit Amsterdam, Amsterdam, Netherlands

**Keywords:** Perception, Sensory processing, Neural encoding

## Abstract

Humans and many other animals possess an innate ability to rapidly perceive numerosity: the number of objects in a visual scene. Numerosity perception is influenced by adaptation, whereby previously viewed numerosities affect perception of the current image’s numerosity. Parietal and frontal neural populations are tuned to specific preferred numerosities, and this tuning is affected by adaptation. A parallel line of research has revealed that early visual responses monotonically increase with numerosity. Here we use ultra-high field (7 T) fMRI to show that these monotonic responses become less pronounced after adaptation to higher numerosities. Moreover, this neural adaptation effect becomes stronger as we progress through the early visual hierarchy (V1-V3, hV4, LO1-LO2 & V3A/B). These findings show that numerosity adaptation has neural effects on the early visual responses as well as the frontoparietal numerosity-tuned responses. Together, these distinct neural effects are consistent with many features of perceptual numerosity adaptation.

## Abstract (web summary)

Numerosity adaptation suppresses monotonic neural responses to numerosity displays in the early visual cortex, with more suppression for higher numerosity adaptors. Therefore, numerosity adaptation effects begin in early sensory stages of processing.

## Introduction

Numerical cognition leverages aspects of perception, attention and working memory to construct a quantitative understanding of the world that eventually allows advanced abilities like mathematics. Numerical cognition’s simplest stages require only an ability to estimate and perceive object number, or numerosity, often called the ‘number sense’. This simple numerosity perception is found in many animals, including humans^1,2^ and non-human primates^3^, but also birds^4^, amphibians^5^, fish^6^, and insects^7^. Numerosity perception may provide a selective advantage in any animal by helping to forage for food, like finding the plant with the most fruits^8,9^. Some have proposed that numerosity perception reflects non-numerical image features that are often correlated with numerosity, like density^10^ or contrast energy at high spatial frequencies^11^. However, extensive recent evidence demonstrates that humans perceive numerosity itself more quickly and accurately than these non-numerical features^12–14^.

Which neural responses underlie numerosity perception? Two broad classes of responses have been described: numerosity-tuned responses and monotonically changing responses. In numerosity-tuned neural populations, the response peaks at a specific (preferred) numerosity and gradually decreases with distance from this numerosity^15^. Numerosity-tuned responses have been found in single neurons in cats^16^, monkeys^3,15^, humans^17^, crows^18^ and chickens^19^. We have also revealed the numerosity tuning of neural populations throughout the human brain by combining ultra-high field (7 T) functional magnetic resonance imaging (fMRI) and neural model-based analyses^20–22^. Numerosity-tuned responses are located in the association cortices of humans^22^ and monkeys^23^. Their responses closely predict numerosity perception across trials^15,24^ and across individuals^25–27^, positioning them as an important basis of numerosity perception^28^.

The second class of neural populations increases their response amplitude monotonically as numerosity increases. Such monotonic responses, sometimes described as summation coding^29,30^, have long been predicted as an intermediate stage in computational models for the derivation of downstream numerosity-tuned responses^30–35^. Monotonically responding populations have been described using EEG and fMRI^36–38^. These responses were found in early visual cortex (including V1^38^, V2, and V3^38,39^) with very short latencies^37^, indicating that these monotonic responses are implicated in the earliest stages of feedforward visual processing. Surprisingly, for such early visual responses, these responses to numerosity are not strongly affected by non-numerical features like item size and spacing^36–38^.

How can such early responses already encompass numerosity information? This may be explained by close relationships between numerosity and aggregate Fourier power in the stimuli used in most experiments^38^. To illustrate, a single V1 neuron can be modelled as a spatial Fourier filter, responding to a particular combination of orientation and spatial frequency. The response of the large neural populations recorded by EEG or fMRI will then aggregate the response power throughout the Fourier spectrum. Aggregate Fourier power follows numerosity closely, with little effect of item size or spacing. Indeed, monotonically increasing responses in early visual areas follow the logarithm of aggregate Fourier power more closely than the logarithm of numerosity^38^. As such, the established response properties of the early visual cortex give a population-level monotonic response to Fourier power from which numerosity can be straightforwardly computed.

In addition to these monotonically responding populations and numerosity-tuned single neurons, monotonically responding single neurons^40^ and numerosity-tuned single neurons that prefer numerosities at the extremes of the tested range have been reported in macaque association cortices^23,41^. It is hard to distinguish monotonically increasing or decreasing responses from maximum or minimum numerosity-tuned neurons, respectively^21,35^. Monotonically responding single neurons may represent an intermediate stage bridging early visual monotonic population responses and the association cortex’s tuned single-neuron responses.

Like many visual features, numerosity perception is affected by adaptation^1^, where perceived numerosity is repelled from previously presented numerosities. For example, after repeatedly viewing a high numerosity, lower numerosities are underestimated. How does numerosity adaptation affect neural responses to numerosity? First, in fMRI repetition suppression paradigms, repeated presentation of a single numerosity suppresses parietal neural responses to similar numerosities more than responses to more different numerosities^27^. This provides evidence for numerosity-tuned responses in human parietal cortex and suggests that adaptation strongly affects numerosity-tuned responses. Similarly, numerosity adaptation strongly reduces the ability to distinguish between the patterns of activity evoked by different numerosities in parietal cortex using multivariate classification methods, suggesting adaptation suppresses or changes the patterns of response to specific numerosities^42,43^. Finally, we have shown that numerosity tuning in neural populations with numerosity preferences near the adaptor is repelled from the adapted numerosity, while that in neural populations with numerosity preferences further from the adaptor is attracted toward the adapted numerosity^44^. This mixed change in numerosity preferences occurs in all numerosity-tuned responses throughout the association cortices and suggests some form of normalization across the whole set of numerosity-tuned responses.

However, it remains unclear whether early visual monotonic responses to numerosity are affected by adaptation and may therefore contribute to later adaptation effects on numerosity-tuned responses. Here, we therefore analysed these early visual monotonic responses in an ultra-high field (7 T) fMRI data set, where we have previously shown numerosity adaptation effects on numerosity-tuned responses^44^. During fMRI scanning, participants viewed the same sequence of changing numerosities (to map numerosity preferences) in three conditions, where this was alternated with a low numerosity adaptor, a high numerosity adaptor and an adaptor matching the sequence of changing numerosities. In the current study, we compared the amplitudes of responses to these conditions in the early visual cortex. Higher numerosities produce a stronger neural response in the early visual cortex than lower numerosities. We therefore hypothesized that adaptation to a higher numerosity would more strongly suppress the monotonic response to subsequently viewed displays, by more strongly reducing the sensitivity of the responsive neural populations.

## Results

### Monotonic responses to numerosity displays in the early visual cortex

**Fig. 1:**
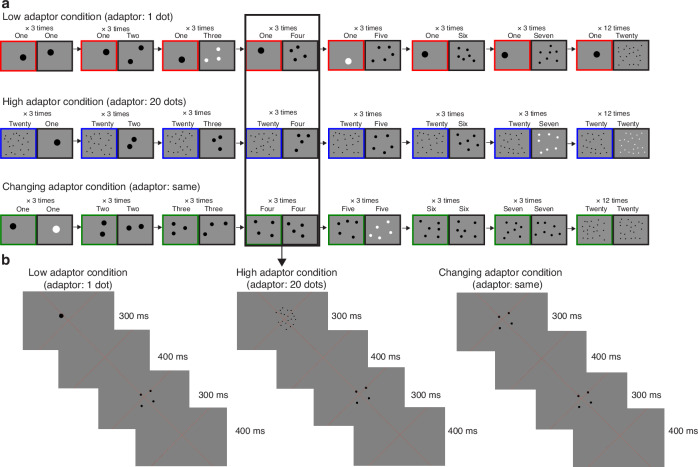
Numerosity stimuli. **a** Schematic description of the numerosity response mapping stimuli shown in the ascending progression of one stimulus cycle. Each fMRI time frame (TR) contained an adaptor numerosity (left, colored border), which differed between conditions, followed by a changing numerosity (right, black border). In all three conditions, the changing numerosities increased from 1 through 7, followed by a baseline of 20 dots. The order of this stimulus sequence, which we used previously, can map the numerosity-responsive maps stably in the neural populations. In the low and high adaptor conditions, the adaptor was constant at numerosities of 1 and 20, respectively. In the changing adaptor condition, the changing numerosities were also shown as the adaptor. In all conditions, the same pair of adaptor and changing numerosities was repeated three times (across three TRs) to ensure strong fMRI responses. **b** Example displays presented in a single TR in each condition.

During fMRI scanning, participants viewed the numerosity arrays while performing an orthogonal task (detecting when white dots were shown instead of black dots) that did not involve numerosity judgements. They viewed sequences of progressively increasing and decreasing numerosities (from one to seven and back) to quantify response amplitudes to different numerosities. This sequence was chosen for its efficiency in quantifying response amplitudes to different numerosities^44^, a necessity given the three scanning sessions required per participant. Alternating increases and decreases in these changing numerosities counterbalance the effects of previous changing numerosities on the response to the current numerosity^21^. These progressively changing numerosity displays were presented in three different adaptor conditions (Fig. 1): (1) Preceded by displays containing one item (low adaptor condition); (2) Preceded by displays containing twenty items (high adaptor condition); (3) Preceded by displays matching the changing numerosity (changing adaptor condition). The changing adaptor condition was used as a control condition to identify responsive locations.

**Fig. 2:**
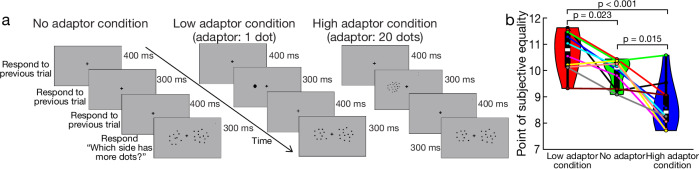
Our stimulus timing induces repulsive behavioural numerosity adaptation. **a** We conducted a behavioural experiment with 10 participants (6 from the fMRI study) to assess the effects of our stimulus sequence outside the scanner in our previous study^44^. A low (1) or high (20) adaptor was presented consistently on one side of fixation. Participants compared a changing test numerosity on the unadapted side to a fixed numerosity reference of 10 on the adapted side. This allowed us to determine the point of subjective equality (PSE), which unadapted numerosity was perceived as matching the adapted numerosity 10. Responses were accepted until the next test display, with no response triggering a repeat trial, maintaining fMRI timing. **b** The PSE was highest in the low adaptor condition, lowest in the high adaptor condition and intermediate in the no adaptor condition. All differences reached significance in paired *t*-tests after Bonferroni correction.

The brief, single presentation of adaptors in our low and high adaptor conditions should elicit numerosity adaptation effects comparable to those in classical behavioural studies for two reasons. First, adaptation effects last for several seconds, allowing prior adaptor presentations to accumulate influence throughout a scan run. To keep a steady state of adaptation in each scan run, we therefore presented the adaptor multiple times before and consistently during each run. Second, behavioural data collected with this dataset^44^ confirm that this specific stimulus timing produces clear repulsive perceptual adaptation effects (Fig. 2), consistent with earlier studies that used similar brief adaptor presentations^45–47^.

**Fig. 3:**
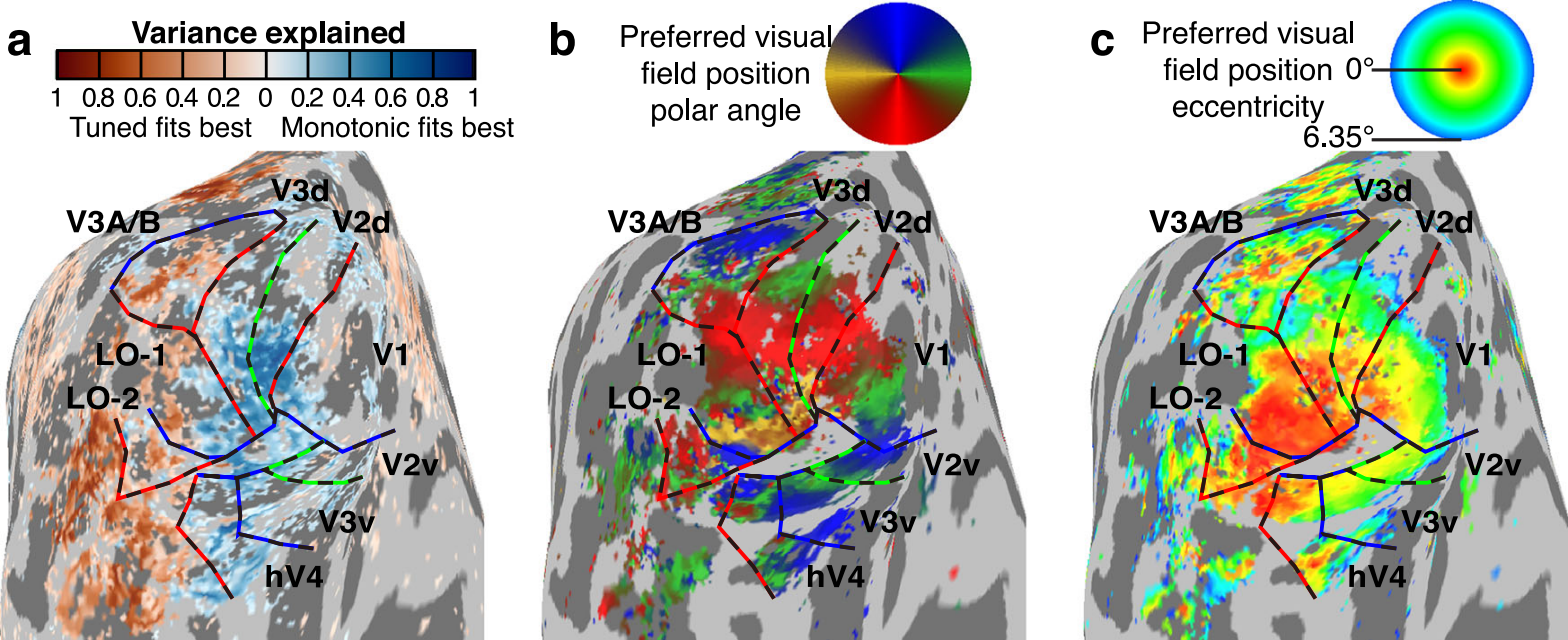
Locations of monotonic responses to numerosity. **a** Blue recording sites show responses that monotonically increased with numerosity (in proportion to the logarithm of aggregate Fourier power), while red recording sites show numerosity-tuned responses. Here, the best-fitting response model explained at least 0.1 (cross-validated *R*^2^) of response variance. See Supplementary Fig. 1a for results from all hemispheres and Supplementary Fig. 1b for results using corresponding monotonic models following log(numerosity). **b** The preferred visual field position polar angle of each recording site (obtained from visual field mapping data) lets us localize visual field map borders at reversals in polar angle progressions. Dashed lines show visual field map borders at the upper vertical meridian (blue), lower vertical meridian (red) and horizontal meridian (green). **c** Each recording site’s preferred visual field position eccentricity. We used this to localize sites with a preferred eccentricity below 1°, whose population receptive fields included the numerosity mapping stimulus area. See Supplementary Fig. 1c, d for visual field maps in all hemispheres.

We used the changing adaptor condition (Fig. 1), without adaptation to a fixed numerosity, to identify responses to changes in numerosity, as we have used this stimulus design in previous studies^21,22,38,44^. We explained responses in all conditions using a monotonic response model where the neural response amplitudes underlying the fMRI response were proportional to the logarithm of the aggregate Fourier power of the changing numerosity display shown during each fMRI time frame. However, we found very similar results (see Supplementary Figs. 1, 2, 4, and 5) in a monotonic response model where the neural response amplitudes were proportional to the logarithm of numerosity, which fit slightly less well than the aggregate Fourier power model^38^. As previously shown^38^, many recording sites in the representation of the central visual field (where the numerosity mapping stimulus was displayed) in visual field maps V1-V3, hV4, V3A/B, LO1 and LO2 showed responses proportional to the logarithm of aggregate Fourier power (Fig. 3). We selected recording sites in each visual field map where preferred visual position eccentricity was below 1°, where a monotonic responses increasing with aggregate Fourier power explained at least 10% of response variance, and where this monotonic response model explained more variance than a numerosity-tuned model.

### Changes in early visual monotonic responses during numerosity adaptation

**Fig. 4:**
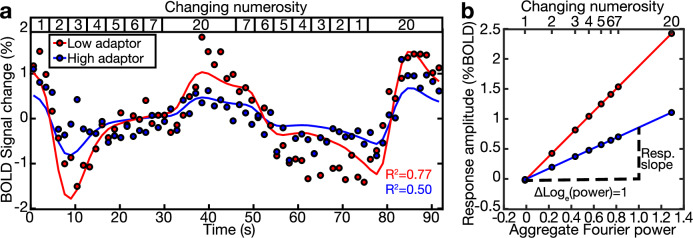
The response of an example recording site (voxel) in V1 to a numerosity mapping stimulus differs between adaptation conditions. **a** As the stimulus’s changing numerosity progressively increased and decreased (top inset), the fMRI BOLD response in both adaptor conditions (colored dots) increased and decreased, after a hemodynamic delay. The responses in both conditions were closely fit by the predictions of the monotonic responses to the logarithm of the aggregate Fourier power of the stimulus (colored lines), scaled with different amplitudes. The range of response amplitudes was greater in the low adaptor condition than in the high adaptor condition. The variance explained (*R*^2^) followed this range of response amplitudes, as a lower amplitude decreases the signal-to-noise ratio of the response. **b** We explained these responses using neural response models in which neural responses monotonically increase proportionally to the logarithm of the aggregate Fourier power of the displays, which follows the logarithm of numerosity closely but nonlinearly^38^. We fit the slope of this proportionality (i.e., the increase in amplitude of the neural response when logarithm of aggregate Fourier power increases by one, ΔLog(power) = 1) using a general linear model. This slope was greater in the low adaptor condition than in the high adaptor condition. See Supplementary Fig. 2 for corresponding monotonic models following log(numerosity).

We first asked whether the change in response amplitudes over the course of a scan in the recording sites differed between the low and high adaptor conditions. The fMRI responses of recording sites in the early visual cortex increased following the aggregate Fourier power (and so the numerosity) of the presented displays in both adaptor conditions (Fig. 4a).

In our response model, the response amplitude of each recording site in each condition was captured by a slope (beta) parameter. This quantified how much the amplitude of the neural response underlying the fMRI signal increased when the logarithm of the aggregate Fourier power of the stimuli increased by one (Fig. 4b). Our response models also included the adaptor stimuli as regressors (see ‘Methods’). This was constant in the low and high adaptor conditions, so it could not explain any response variance.

**Fig. 5:**
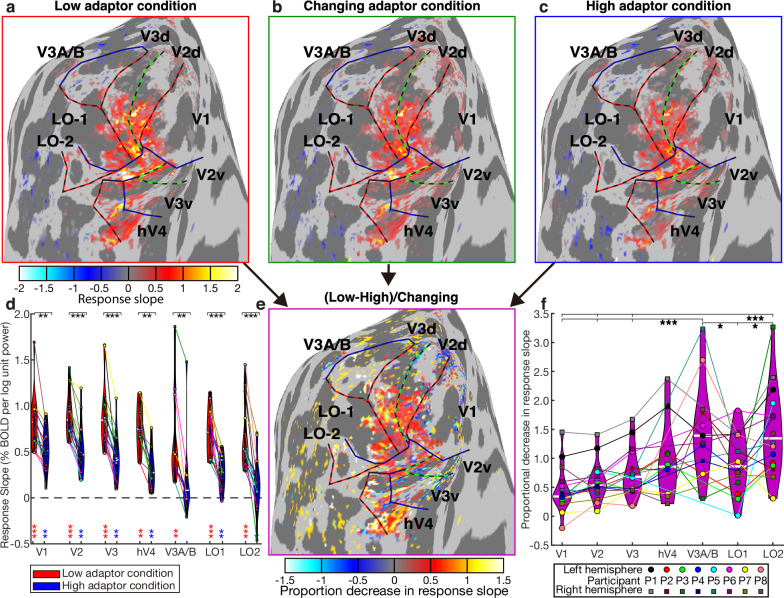
Neural adaptation of monotonic responses increased through the visual hierarchy. **a**–**c** The fMRI BOLD responses increased monotonically with aggregate Fourier power in recording sites throughout the central visual field representations of the early visual field maps, in the low (**a**), changing (**b**), and high (**c**) adaptor conditions. In all conditions, voxels showing a monotonic response in the changing adaptor condition were selected for further analysis. See Supplementary Fig. 3a–c for results from all hemispheres, Supplementary Fig. 4a–c for corresponding monotonic models following log(numerosity) and Supplementary Fig. 5a–c for results from all hemispheres for corresponding monotonic models following log(numerosity). **d** In the average across the recording sites in each visual field map of each hemisphere (*n *= 16 hemispheres), the slope of the monotonic response increase with the logarithm of aggregate Fourier power was significantly positive in both conditions and almost all visual field maps (colored stars, Supplementary Table 1). This slope was greater in the low adaptor condition than the high adaptor condition (black stars show comparisons between conditions in each visual field map, Supplementary Table 2) *p < 0.05, **p < 0.01, ***p < 0.001. Colored markers (linked with colored lines) show the mean in the visual field map example in each hemisphere and condition. See Supplementary Fig. 4d and Supplementary Tables 3 and 4 for corresponding monotonic models following log(numerosity). **e** To compare this reduction in monotonic response amplitude between visual field maps, we calculated the reduction in response slope between low and high adaptor conditions in each recording site, as a proportion of the slope in the changing adaptor condition. See Supplementary Fig. 3d for results from all hemispheres, Supplementary Fig. 4e for corresponding monotonic models following log(numerosity) and Supplementary Fig. 5d for results from all hemispheres for corresponding monotonic models following log(numerosity). **f** This proportional decrease in response amplitude from low to high adaptor conditions (i.e., the neural adaptation effect strength) became greater through the visual processing hierarchy (Supplementary Table 5). Visual field maps marked with brackets to the right of the stars showed significantly stronger proportional decreases than those with brackets to the left of the stars (Supplementary Table 6). See Supplementary Fig. 4f and Supplementary Tables 7 and 8 for corresponding monotonic models following log(numerosity).

To summarize the response change in each visual field map, we first calculated the response slope in each adaptor condition for recording sites throughout the early visual cortex (from V1 to LO2 and V3A/B) (Fig. 5a–c). Notably, some later visual field maps (LO1, LO2, V3A/B) include recording sites with both monotonic and tuned responses: our analyses included only recording sites whose responses are better explained (better fit under cross-validation) by our monotonic response model in the changing adaptor condition. For each visual field map, we took the average slope in each condition across these recording sites in every hemisphere and used these hemisphere averages for statistical comparisons. A two-sided Wilcoxon signed rank test showed that the slope was significantly above zero in both adaptor conditions in all visual field maps except the high adaptor condition in V3A/B (Fig. 5d and Supplementary Table 1). So both conditions yielded monotonically increasing responses to aggregate Fourier power (and so to numerosity). In each visual field map, this slope was significantly greater in the low adaptor condition than the high adaptor condition (Fig. 5d and Supplementary Table 2), a greater neural adaptation of monotonic responses to higher numerosities. We did not find any difference between the constant baseline response amplitudes during any adaptor conditions in any visual field map.

### Progressive increases in adaptation effects through the early visual hierarchy

We then compared the strength of this neural adaptation effect between visual field maps. We first computed a measure of relative slope change; specifically, we subtracted the slope in a high adaptor condition from the slope in a low adaptor condition, and divide by the slope of the changing adaptor condition (Fig. 5e). This metric reflects how much shallower the slope (of responses to increasing numerosities) becomes when preceded by a high compared to a low adaptor, quantifying the relative strength of the neural adaptation effect in each visual field map. An ANOVA (with visual field map as a fixed factor and participant as a random factor) found significant differences between maps in this proportional reduction in response slope (*F*(6,86) = 11.4, *p* = 2.5 × 10^-9^). Post-hoc multiple comparisons revealed a progressive increase in the strength of the neural adaptation effect from earlier to later visual field maps (Fig. 5f; Supplementary Tables 5 and 6). Therefore, the effect of numerosity adaptation on neural response amplitudes increased through the early visual hierarchy.

### Relationships between neural adaptation effects in different brain areas

Finally, in exploratory analyses, we investigated relationships between neural adaptation effects in different brain regions. We found a strong correlation between hemispheres in the proportional reduction of response slope across V1, V2, V3, and hV4 (Supplementary Figs. 6a and 7a). This suggests a strong connection between adaptation effects in these early visual field maps, though it could also reflect general individual differences in fMRI BOLD response amplitude. However, no significant correlations emerged between these early visual maps and later ones (LO1, LO2, V3A/B), nor among the later maps themselves. This might indicate independent effects at these later processing stages or simply more variable effects.

We also explored whether the strength of early visual monotonic effects correlated with the strength of numerosity-tuned response effects, given that tuned responses might be derived from monotonic responses. We tested for a correlation between early visual suppression (proportional reduction in response slope) and the reduction in explained response variance of the tuned response model between low and high adaptor conditions in the numerosity maps. We observed marginal correlations specifically between the numerosity map NTO and visual field maps V1-hV4 (Supplementary Figs. 6b and 7b). However, most of these few significant correlations did not survive correction for multiple comparisons, and no significant correlations were found among other tested visual field or numerosity maps. We tested for a correlation between early visual suppression and the changes in numerosity preferences of the tuned responses between low and high adaptor conditions in the numerosity maps. While comparing numerosity preferences to early visual response suppression was complex due to bidirectional shifts in numerosity preferences^44^, some marginal correlations again appeared between NTO and V1-hV4. (Supplementary Figs. 6b and 7b). In short, these between-hemisphere correlations of monotonic and tuned effects were underpowered and are complicated by notable interhemispheric differences within participants in numerosity maps beyond NTO^22^.

## Discussion

In the current study, we asked whether numerosity adaptation affects the responses of the early visual cortex. First, we found that the monotonically increasing neural response to numerosity occurred regardless of numerosity adaptation, from V1 through the early visual hierarchy to V2, V3, hV4, V3A/B, and LO1-LO2. Second, in all these visual field maps, the amplitude of this monotonic increase (slope) was reduced when the adapting numerosity was higher. Third, the proportion by which the response slope was reduced during higher compared to lower numerosity adaptation (i.e., the magnitude of the adaptation effect) increased hierarchically from V1 onward. Fourth, the magnitude of this adaptation effect generally correlated among the visual field maps V1, V2, V3 and hV4.

In this study, we focus on the early visual neural response that monotonically increases with numerosity^36–38^. We have explained these findings by the close relationship between numerosity and contrast energy in the spatial frequency domain^38^. Image contrast refers to the overall variation in brightness or color distribution across the entire image, typically reflecting the range of luminance values within the image. In the spatial frequency domain, this variation is measured as contrast energy, expressed in Fourier power. At a fixed image contrast, this aggregate Fourier power follows numerosity closely but nonlinearly, with little effect of size or spacing, and predicts population responses in V1 and computational models^32,33,35^ more closely than numerosity does^38^. Recent modelling studies also show how image filters specifically modelling the properties of early visual neurons could capture these contrast properties to yield early visual monotonic responses^48^. This provides a response in the early visual cortex from which numerosity itself may be straightforwardly derived. Indeed, responses in the numerosity-tuned populations of the association cortices are more closely predicted by numerosity than aggregate Fourier power^38^. Therefore, we describe the monotonic responses in the current study as responses to contrast and the tuned responses as responses to numerosity. However, we found a very similar pattern of results if we model the early visual responses as functions of the log(numerosity) (Supplementary Figs. 1b, 2, 4, 5 and 7), rather than as functions of the logarithm of the aggregate Fourier power of our displays.

Adaptation effects on numerosity perception^1^ have always been assumed to reflect changes in the responses of numerosity-tuned neurons. This is a reasonable assumption for several reasons. First, when the adaptation of numerosity perception was first described, numerosity-tuned neurons had recently been found in macaque parietal and frontal cortices^3,23^, and tuned effects of repetition suppression were found in human parietal cortex^27^. Then, the monotonic response was shown in the parietal lobe^40^ and early visual monotonic responses to numerosity were only described years later^36,37^. Second, adaptation effects are often found for image features with tuned neural representations, like orientation^49^ and motion direction^50^. Third, adaptation to a low numerosity has been shown to increase perceived numerosity^1,51^, as well as adaptation to high numerosity decreasing perceived numerosity. The bidirectionality of this repulsive effect seems likely to reflect effects on numerosity-tuned neural populations with different numerosity preferences. Specifically, adaptation to a numerosity below the numerosity preference of a numerosity-tuned neuron should suppress responses to lower numerosities more than responses to higher numerosities. This should thereby increase the numerosity yielding the largest response (the numerosity preference)^28^. Accordingly, we have recently shown (using the present data set) that tuned neural numerosity preferences are affected by adaptation^44^.

However, converging evidence also suggests that the neural effects of perceptual numerosity adaptation begin at early visual processing stages, with spatially specific responses to image contrast. First, perceptual numerosity adaptation is highly spatially specific (limited to the adapted location)^1^, while numerosity-tuned neurons have large spatial receptive fields and their response to numerosity does not depend on the stimulus falling within that receptive field^22,38,52,53^. Second, perceptual numerosity adaptation is weaker when the adaptor and test displays differ in color^54^ or other low-level visual features^55^. Different low-level features activate distinct neural populations in early visual processing, so an adaptation effect on the population responding to one feature is unlikely to affect populations responding to other features. Conversely, similar numerosity-tuned responses are found regardless of item color^56^, so adaptation effects working on these populations should generalize across low-level features. Third, compelling recent results^57^ show that perceived numerosity is affected by adaptation to gratings with no numerosity but a spatial frequency matching that of the numerosity display. Fourth, recent results show that the strength of the numerosity adaptation effect is greater when the positions of the individual items in the adaptor and test displays overlap^58^. Again, different positions activate distinct neural populations in early visual processing, but similar numerosity-tuned responses are found regardless of item position^22,38,52,53^. Fifth, the increase in perceived numerosity after low numerosity adaptation is far weaker than the decrease after high numerosity adaptation^51,58^. The asymmetry of this bidirectional effect may reflect an additional effect of adaptation at the monotonic response stage for high numerosity displays. Finally, the numerosity adaptation effect becomes weaker as contrast decreases^1^, though it remains clear even at low contrasts. Together with the present results, these results suggest that perceptual numerosity adaptation at least partly originates in early visual processing stages with spatially specific responses to contrast.

Importantly, none of these findings show that perceptual numerosity adaptation arises only through early visual contrast adaptation and indeed several results speak against this interpretation. First, we found that effects on monotonic responses become progressively stronger through the early visual hierarchy, suggesting additional neural numerosity adaptation effects at many stages of numerosity processing. Second, recent results^59^ show that responses to numerosity in more anterior areas of the association cortices depend progressively more on the context of recently presented numerosities. Third, the effects on monotonic responses that we see are only correlated with effects on tuned responses in the most posterior numerosity map. All of these results suggest progressively increasing neural adaptation effects throughout the numerosity processing hierarchy, not effects at an early stage alone. Furthermore, adaptation effects on visual numerosity perception can also be produced by adapting to quantities in other sensory modalities^60–62^, though these cross-modal adaptation effects are weaker than the effects of adaptation to visual numerosity itself. Finally, beyond adaptation, numerosity estimation is reduced when individual items are connected by bars^63^. This effect is not present in the earlier visual responses to numerosity^39^ and cannot be explained by changes in the spatial frequency domain contrast of the displays^38^, so at least some effects on numerosity perception depend on later stages. We therefore propose that neural effects at many stages of numerosity processing contribute to perceptual numerosity adaptation effects. Neural populations in many areas represent information about numerosity in either their monotonic or tuned responses, with hierarchical processing of each response across many stages^22,38^ and tuned responses likely being derived from monotonic responses^30,32^. As adaptation may be best understood as a property of all neural responses, we can expect adaptation effects at all of these stages, with effects at one stage likely being inherited by the next.

Our results do not convincingly demonstrate that adaptation effects on early visual monotonic responses ultimately cause adaptation effects on numerosity-tuned responses. Indeed, it is not yet clear that early visual monotonic responses are required to produce a numerosity-tuned response. Nevertheless, several findings suggest that adaptation effects on numerosity-tuned responses are inherited in part from effects on early visual contrast representations. First, almost all visual inputs to the cortex come through the primary visual cortex, which represents image features by contrast-driven responses in the spatial frequency domain. There is no other pathway through which numerosity-tuned neurons could be activated by visual stimuli. Second, computational models for the derivation of numerosity-tuned responses^30–32,34^ generally rely on an intermediate stage with monotonic responses to numerosity. We have previously shown that the monotonic responses to numerosity shown by two very different neural network models^32,33^ are better predicted by early visual responses to contrast^38^. Changing the early visual contrast representation seems likely to change any response derived from this representation.

As such, our findings of multi-level neural adaptation impact on the current understanding of numerosity processing in three crucial ways. First, while numerosity processing is often considered a high-level cognitive function linked to mathematics^64–66^ and decision-making^67^, with neural adaptation correspondingly shown in association cortices^44^, recent studies increasingly point to lower-level processes^32,33^ in early visual cortices^37,38,48^. Our work confirms this by revealing neural numerosity adaptation effects at these lower levels. Second, we demonstrate that neural adaptation effects accumulate hierarchically through multiple stages of early visual processing. This suggests that numerosity adaptation may build on (rather than being solely explained by) effects on early visual contrast processing. Finally, by suggesting that perceptual numerosity adaptation may be partly rooted in early visual mechanisms, our study offers a potential neural basis for recent behavioural findings demonstrating the influence of low-level visual features on perceptual numerosity adaptation^54,55,57,58^.

We have previously used this data set to reveal numerosity adaptation effects on the numerosity preferences of numerosity-tuned neural populations in the parietal, frontal and lateral occipital lobes^44^. We tested whether the strength of this tuned neural adaptation effect was correlated with the strength of the monotonic adaptation effect described here. Unfortunately, this analysis (Supplementary Fig. 6b, c) lacked the statistical power to show such correlations because the data set only included eight participants or 16 hemispheres. Some trends suggest that hemispheres with larger reductions in monotonic response slope in V1-hV4 may also show greater suppression of tuned responses and larger changes in numerosity preferences between high and low adaptor conditions, specifically in numerosity map NTO. Beyond NTO, the tuned numerosity preferences exhibit a strong hemispheric lateralization^22^, which was not found in the monotonic early visual responses.

All our conditions only present the adaptor very briefly (and typically once) before each presentation of a changing numerosity, although many times over different presentations of changing numerosities. Is this sufficient to produce repulsive numerosity adaptation effects in perception? Or does this instead produce attractive serial dependence effects that occur when single presentations of a particular numerosity bias perception of the numerosity in the next presentation^68,69^? We have previously shown that the stimulus timing used here produces a clear repulsive adaptation effects^44^. Previous results also showed repulsive adaptation effects with brief adaptor presentations^45^. Again, here, these brief but frequent presentations, although separated by changing numerosities, would be expected to affect the average level of recent activity in the early visual cortex that we propose underlies the effects we observe.

Demonstrating perceptual adaptation with our stimulus timing requires participants to attend to the test numerosity, though not necessarily the adaptor. While judging the test numerosity necessitates attention to numerosity, which might influence its perception, our fMRI participants attended the items without judging the changing numerosities. Attention to numerosity itself enhances tuned neural responses to numerosity^65^, but we have repeatedly shown responses to numerosity without attention specifically to numerosity. Attention to numerosity may potentially enhance early visual response amplitudes, so adding explicit attention to numerosity might strengthen the effects we describe, but we do not expect it to qualitatively change these effects.

While we have previously shown that a lack of any attention to the dots (in a non-numerical task) strongly affects responses to numerosity^56^, our fMRI participants did attend to the dots in a non-numerical task. Although neither our behavioural nor fMRI experiments required attention to the numerosity of the adaptor, they may differ in the attention allocated to the dots or their location. In our fMRI data, adaptors were always presented at fixation, and we compared the effects of the different adaptors. However, in our behavioural experiment, an adaptor was presented on one side of the screen with nothing on the other side. In this situation, the appearance of the adaptor on one side only is likely to implicitly attract attention to the adaptor location and numerosity^70,71^ and so increase the neural response to the adaptor and its adaptation effects. Therefore, perceptual adaptation effects in our fMRI experiment may be weaker than in our behavioural experiment. Nevertheless, in our fMRI experiment, the appearance of the adaptor alone and its presentation at fixation are also likely to attract attention. Further work would be needed to address how attention affects neural numerosity adaptation effects.

Functionally, adaptation is usually proposed to adapt perception to the context of recently seen sensory stimuli, thereby increasing sensitivity in the stimulus range we are currently working with by increasing discriminability around the adapted range^72^. Seeing contrast adaptation as a fundamental contributor to numerosity adaptation instead suggests numerosity adaptation’s functional role may be to help separate numerosity from contrast. Both numerosity and the contrast between items and their background (i.e., item contrast) similarly affect the image’s total Fourier power in the spatial frequency domain (i.e., image contrast)^38^. An image can have greater total Fourier power because it contains more items or greater item contrast. To determine numerosity, we need to normalize the image contrast for item contrast. Indeed, responses in V1 are strongly contrast-dependent, while responses in the first areas showing numerosity-tuned responses (visual field maps TO1 and TO2, i.e., area hMT+) are minimally affected by item contrast^73^. Therefore, under normal circumstances, contrast adaptation may serve to normalize item contrast by considering the contrast of recently viewed items, and thereby yield a contrast-invariant representation to numerosity. However, during the unusual circumstances of numerosity adaptation, numerosity affects image contrast while item contrast is held constant. This may thereby disrupt this normalization process, leading to inaccurate numerosity perception. This view sees mechanisms of numerosity adaptation as inherent to the process of numerosity estimation itself, rather than an adaptive aspect of numerosity perception. These views are not mutually exclusive.

## Conclusions

The current results show a central role for early visual cortex in the neural basis of numerosity adaptation, increasing in strength through the visual processing hierarchy. This shows that adaptation to higher-level features can indirectly reflect effects in early sensory processing, in this case, early visual contrast representations. These early visual effects may be inherited by later numerosity-tuned neural populations, with separate neural adaptation effects also likely acting in numerosity-tuned stages. Therefore, the neural basis of numerosity adaptation likely involves effects at all levels of numerosity processing. Together, these pervasive neural effects throughout the brain seem likely to underlie the strong and multifaceted perceptual effects of numerosity adaptation.

## Methods

### Participants

We recruited eight human participants (five male, three female; age range 26–52 years). One was left-handed. All were well educated, with good mathematical abilities, and had normal or corrected-to-normal visual acuity. All gave written informed consent. None were excluded. All experimental procedures were approved by the ethics committee of University Medical Centre Utrecht (protocol number 09/350). All ethical regulations relevant to human research participants were followed.

### Numerosity stimuli

We used MATLAB (MathWorks, Inc.) and the Psychophysics Toolbox^74,75^ to generate and present experimental stimuli similar to our past studies^21,22,44,52^. The numerosity stimuli were presented on a 69.84 × 39.29 cm LCD screen (Cambridge Research Systems) positioned behind the MRI bore. Participants were required to lie still and view the display through a mirror attached to the head coil. The total distance from the attached mirror to the display screen was 220 cm, and the display resolution was 1920 × 1080 pixels.

Two large, thin and red cross lines were presented in the entire display to aid accurate fixation at the cross intersection in the centre of the display. All items in the numerosity stimuli were positioned pseudo-randomly and limited within a circle centred on the fixation of 0.75° of visual angle (radius), minimizing the extent of the numerosity pattern, allowing it to be viewed without eye movements, and falling within the population receptive field of the fMRI recording site responding to the central visual field. The pseudo-random positions of these items were constrained so that items were evenly spaced throughout this limited circle, avoiding perceptual grouping. Each numerosity stimulus presentation contained a new pseudo-random dot pattern. We kept the total surface area of all display items constant regardless of numerosity, so that display luminance was unaffected by numerosity.

In all conditions, the numerosities 1 through 7 and 20 were presented as dots on a gray background (Fig. 1). Each numerosity stimulus was presented briefly (300 ms) to ensure participants had no time to sequentially count the dots. The numerosity stimulus was followed by an interstimulus interval (ISI) of 400 ms, showing a uniform gray background, then the next numerosity stimulus. In each 1400 ms (one fMRI volume acquisition, TR), we first showed an adaptor numerosity, which differed between the three conditions, then a changing numerosity, which was the same for all three conditions. The changing numerosities varied from 1 through 7, with a baseline of 20 dots. For the changing numerosities 1-7, this was repeated three times (across three TRs) before the numerosity changed, to ensure strong fMRI responses and allow enough time to distinguish the hemodynamic responses to different numerosities. Previous results have shown that a short stimulus timing and a predictive stimulus series can produce a repulsive adaptation effect^44,45^. When the changing numerosity was 20, this was repeated 12 times (across 12 TRs) to better distinguish between numerosity-tuned and monotonic responses. A monotonically increasing response to numerosity should have a high amplitude during this period. However, a tuned response with a numerosity preference far below 20^20^ should have a lower amplitude during this period because a numerosity of 20 dots should be well outside of the range that elicits strong responses. This also allowed us to distinguish neural populations with very small tuning widths, which never responded to the changing numerosities 1 through 7, and populations with very large tuning widths, which always responded to these numerosities^21^.

In the low and high adaptor conditions, the alternating adaptor numerosity was held constant at 1 and 20, respectively. In the changing adaptor condition, the same numerosities were shown in the adaptor as the changing numerosity.

The changing numerosity stimuli were first presented in ascending order (1 to 7) for 4.2 s (3 TRs) each, next followed by 16.8 s (12 TRs) where the stimulus contained 20 dots, then followed by the numerosities in descending order (7 to 1) for 4.2 s (3 TRs) each, finally followed by another same long period of 20 dots. Alternating increases and decreases in these changing numerosities counterbalance the effects of previous changing numerosities on the response to the current numerosity^21^. This sequence was repeated four times in each scanning run, resulting in a run duration of 369.6 s. Therefore, each of the changing numerosity stimuli 1 through 7 was shown for a total of 24 times in each functional run. In the changing adaptor condition, these changing numerosities were also shown as the adaptor, adding another 24 times in each run.

The dots showing both the adaptor and changing numerosities were shown in black in 90% of dot presentations, while in the remaining 10%, the dots were shown in white (Fig. 1). Participants were instructed to press a button when the dots were shown in white instead of black (which is very easy at all numerosities) to ensure that they were paying attention to the stimuli during fMRI acquisition. No numerosity comparisons were required, because numerosity comparisons are more difficult at high numerosity, making it impossible to distinguish the effects of numerosity and task difficulty in fMRI designs with numerosity comparisons.

### Behavioural validation of numerosity adaptation

We next describe the results of an existing behavioural experiment, in which we previously showed reliable behavioural adaptation effects, using a similar—single adapter—experimental design. Ten participants (six also in the fMRI experiment) were tested on a MacBook Pro, seated 60 cm from the 32 × 29 cm, 1280 × 800, 60 Hz display. Adaptors were circular patches (7° diameter) of 1 (0.6° dot diameter) or 20 dots (0.15° dot diameter). The reference was a 10-dot patch (0.2° dot diameter). Dots were randomly scattered without overlap within patches, centred 8° left or right of fixation (100 trials total). In adaptation conditions, a 300 ms adaptor was followed by a 400 ms ISI. The 300 ms reference then appeared at the adaptor’s location, while the test patch, whose numerosity varied via a Minimum Expected Entropy staircase, appeared contralaterally. Midway through the staircase, adaptor and reference locations swapped sides.

Participants fixated centrally and performed a 2AFC task: responding to which patch had more dots as quickly and accurately as possible, and guessing if unsure. To mirror our fMRI paradigm’s timing, trials progressed without waiting for a response. Participants could respond any time before the next test stimulus, and the trial was repeated if they did not respond.

The resulting data were binned by test numerosity, and the proportion of trials judged more numerous was calculated in each bin. We fit a cumulative Gaussian function to estimate the PSE for each condition in each participant. We then ensured that paired differences between PSEs in different conditions were normally distributed (Shapiro-Wilk test, *p* > 0.05) and performed paired t-tests between each pair of conditions, using Bonferroni correction for the three comparisons performed.

### Visual field mapping stimuli

In a separate scanning session, visual field mapping was used to delineate visual field maps and determine the position selectivity of our recording sites, following protocols described previously^38,76,77^. Briefly, a bar filled with a moving checkerboard pattern stepped across a 6.35° (radius) circle in the display centre in eight (cardinal and diagonal) directions. Participants fixated on the same central fixation cross, pressing a button when this changed color to ensure fixation and attention.

### MRI acquisition

We acquired MRI data on a 7 T Philips Achieva scanner for a previously published study^44^. Similar acquisition protocols are described fully in other previous studies^22,52^. Briefly, we acquired T1-weighted anatomical scans, automatically segmented these with Freesurfer (http://freesurfer.net), then manually edited labels to minimize segmentation errors using ITK-SNAP (http://www.itksnap.org/). This provided a highly accurate cortical surface model at the grey-white matter border to characterize the cortical organization of the measured responses.

Functional T2*-weighted 2D echo planar images were acquired using multiband acquisition (multiband factor: 2) and anterior-posterior encoding, and a 32-channel head coil, at a resolution of 1.77 × 1.77 × 1.75 mm, with a field of view of 227 × 227 × 70 mm. The TR was 1400 ms, the echo time (TE) was 25 ms, and the flip angle was 70°. Functional runs were each 273 time frames (382.2 s) in duration, of which the first 9 time frames (12.6 s) were discarded to ensure the signal was at steady state. Moreover, in each session, we acquired a top-up scan in the same position and resolution with the opposite phase-encoding direction to correct for image distortion in the gradient encoding direction^78^.

Three scanning sessions were required for each participant. In each scanning session, 3 functional runs were acquired for the changing adaptor condition (9 runs in total, total duration: 57 min 20 s) and 3–4 runs for the low and high adaptor conditions (in total 10 runs each for these adaptor conditions in total, total duration: 63 min 42 s; with the exception of one participant where 9 runs were acquired for each condition due to technical issues). The additional run we acquired for the low and high adaptor conditions helped ensure strong fMRI responses, because the changing numerosity stimuli were presented less frequently due to the interleaved adaptor stimuli. The order of the conditions was counterbalanced across runs within and between participants.

### fMRI preprocessing

The functional data were co-registered to the anatomical space using AFNI (afni.nimh.nih.gov;^79^) as described previously^38,44,80^. A single transformation matrix was constructed, incorporating all the steps from the raw data to the cortical surface model, to reduce the number of interpolation steps to one. For the fMRI data, we first applied motion correction to the functional data (3dvolreg). We also applied motion correction to the images that were acquired using opposing phase-encoding direction, then determined the distortion transformation between these and the functional runs (3dQwarp) to correct for spatial distortions in the functional scans (3dNwarpApply). Then we determined the transformation that co-registers this functional data to the T1 with the same resolution, position and orientation as the functional data (3dvolreg). We finally determined the transformation from this T1 image to a higher resolution (1 mm isotropic) whole-brain T1 image (3dUnifize, 3dAllineate). We applied the product of all these transformations for every functional volume to transform our functional data to the whole-brain T1 anatomy. We repeated this for each fMRI session to transform all their data to the same anatomical space. We then imported these data into Vistasoft’s mrVista framework (github.com/vistalab/vistasoft) for analysis and model fitting, which relied on custom MATLAB scripts. For each adaptor condition, the time series of separate scans was then averaged together, resulting in a very high signal-to-noise ratio.

### fMRI data analysis

#### Neural response models for responses to numerosity

For each fMRI recording site (voxel), we interpret the fMRI responses to the numerosity stimuli using two neural response models: a numerosity-tuned population receptive field (pRF) model^21,22,52,76^ and a monotonic response model^36–38^. These each describe the recording site’s response using a small set of parameters that we can then compare between adaptor conditions.

For the monotonic response model, the predicted neural response at each recording site is proportional to the logarithm of the aggregate Fourier power (in the spatial frequency domain) of the displays with each numerosity^38^, shown at each time point. We convolved this neural response time course with an HRF to give an fMRI response time course prediction. In each adaptor condition, we used a general linear model to compare this prediction to the fMRI response time course at each recording site. This determined the slope of the relationship between the prediction and the response (proportional to the neural response amplitude, following a positive or negative relationship), together with the response variance explained by this scaled prediction. As we have previously shown^38^, this contrast-driven response model is closely but nonlinearly related to a monotonic response to the logarithm of the presented numerosity in each display. However, it predicts the responses of the early visual cortex and neural network to numerosity displays more closely than numerosity does. We also repeated our analyses using a model describing a monotonic response to the logarithm of the presented numerosity at each time point, giving very similar results.

The numerosity-tuned pRF model describes the aggregate tuning of neural populations in each record site using a logarithmic Gaussian function with two free parameters: preferred numerosity (mean of the Gaussian function) and tuning width (standard deviation of the Gaussian in logarithmic numerosity space). We started by generating a large candidate set of combinations of preferred numerosity and tuning width. Preferred log numerosities ranged from 0.007 (i.e., log(1.007)) to 5.491 (log(242)) in steps of 0.01, while log-space tuning widths (standard deviations) ranged from 0.03 to 3 in steps of 0.0074. Gaussian functions with a log preferred numerosity more than two standard deviations above 2.64 (log(14)) were not tested, as these only predict responses to numerosity 20. For every candidate combination, we predicted a neural response time course as the amplitude of the candidate neural response function at each time point’s presented numerosity. We then convolved this candidate neural response time course with a hemodynamic response function (HRF), giving a corresponding candidate fMRI response time course prediction. For each fMRI recording site and stimulus condition, we chose the fMRI response time course prediction that most closely followed the recorded response time series (by minimizing the sum of squared errors between the predicted and observed fMRI time series). We then took the parameter combination that generated this fMRI response time course prediction, together with the goodness of fit of this prediction, for further analysis. We quantify this goodness of fit as the variance explained by the model, i.e., *R*^2^, the proportion of the variance of the fMRI response time course that is outside the residual of the fit model.

In modelling the responses to the three adaptor conditions, we fit models that included the aggregate Fourier power (or numerosity) of both the adaptor display and the changing numerosity display when making the predictions of fMRI responses. Specifically, the sequence of presented adaptor stimuli, convolved with the hemodynamic response function, was a distinct regressor. However, the stimulus was designed so that models that include the adaptor would produce closely related predictions and parameter estimates to models that do not, and we confirmed that our results were unaffected by this choice. In the high and low adaptor conditions, the adaptor presented a constant numerosity throughout the run. This produces a constant response throughout the run in both the monotonic and tuned response models. In general linear modeling frameworks, this adaptor then adds a constant regressor to the predicted response. FMRI data has an arbitrary baseline that is anyway captured by another constant component (which we do not analyse), so any further constant component contributes to that baseline without affecting other model parameters. In the changing adaptor condition, the adaptor presented the same numerosities as the changing numerosity that our models’ responses follow, so the regressor following this changing adaptor was identical to the monotonic response model’s regressor. In general linear models, before scaling factors are fit, this doubles the amplitude of the predicted response to the changing numerosity. This therefore halves the fit scaling between the fMRI response and the predicted response to the changing numerosity display, compared to a model that does not include a regressor for the response to the adaptor display. We note here that simply doubling the strength of the modeled stimulus reflects an oversimplified (linear) model of fMRI response accumulation. This only affects our calculation of the proportion by which the high adaptor condition suppresses responses, which would simply be halved if the adaptor stimulus were not included in our response model. This has no effect on the statistics. Any changes in fMRI responses between adaptor conditions can only arise through non-linear interactions between the response to the adaptor and the changing numerosity stimuli.

It is important for further analyses to distinguish between monotonically increasing and numerosity-tuned responses. We used the changing adaptor condition to identify responses to changes in numerosity, as we have used this stimulus design in previous studies^21,22,38,44^ and it maximizes neural response amplitude and the goodness of fit of our models. We fit both monotonic and tuned models to the averages of the odd and even numbered scans in this condition. We then evaluated the response predictions of both resulting models on the complementary half of the data (i.e., cross-validation) because the tuned model is fit from a larger set of predictions, which follow more complex functions. During this evaluation, we allowed the response predictions to rescale in amplitude (but not change sign) between fitting and evaluation because the complementary halves of the data were often acquired in different scanning sessions, which can arbitrarily differ in fMRI signal amplitude. We then computed the residual sum of squared errors between the responses and predictions across both halves and for each voxel chose the model with the lower residual.

A numerosity-tuned response can be clearly identified when the preferred numerosity is within the range of the changing numerosities 1 through 7, because this shows the response amplitude decreases for higher numerosities. Therefore, our numerosity-tuned pRF models make and test predictions outside of this range to show that preferred numerosity estimates within this range predict responses better than functions with a preferred numerosity outside of this range. A monotonic response can be clearly identified when a monotonic response model fits better than a numerosity-tuned model. However, voxels that fit slightly better by a numerosity-tuned model with a numerosity preference above 7 are also likely to reflect monotonic responses, because our previous experiments using a larger numerosity range demonstrate that very few voxels show numerosity-tuned responses with preferences above 7^20^. We therefore also use monotonic models of voxels where the numerosity-tuned model estimates a numerosity preference above 7.

Moreover, we also exclude from further analysis of numerosity-tuned pRF models the recording sites for which the response models in the changing adaptor condition explained less than 0.2 of the response variance.

### Neural response models for visual field position and definition of visual field maps

We localized monotonic responses to the area around the occipital pole, the location of the visual field maps of the early visual cortex^36–38^. We therefore asked how adaptation affects the localization of monotonic responses in these early visual field maps. We fit the responses to the visual field mapping stimuli using a standard visual spatial pRF analysis^76,77^. We defined visual field maps' borders based on the reversals in the cortical progression of the polar angle of voxels’ visual field position preferences, manually identifying these on an inflated rendering of each participant’s cortical surface^38,64^. These formed our main regions of interest. As well as the early visual field maps (V1, V2, V3, hV4), we also identified mid-level visual field maps (LO1, LO2 and V3A/B), which showed monotonically responding recording sites in some hemispheres.

### Statistics and reproducibility

In order to quantify the change in monotonic response amplitudes between different adaptor conditions, we analysed the parameters of monotonic models fit to the responses of recording sites in each of the early visual field maps (V1-V3, hV4, V3A/B, LO1, LO2) in each hemisphere (*n* = 16 hemispheres). Specifically, we compared the slope of the relationship between the monotonic response prediction and the recorded response, i.e., the increase in amplitude of the neural response underlying the fMRI signal when the aggregate Fourier power of the changing numerosity display increases by one (Fig. 4b). We also repeated this using a log(numerosity) response model, where the slope parameter reflects the increase in amplitude of the neural response underlying the fMRI signal when the logarithm of the presented numerosity increases by one. This gave very similar results.

To make these comparisons between monotonic responses in the different adaptor conditions, we first take all the recording sites within a visual field map and extract their preferred visual field positions from the visual field position response models. For each recording site, we then extracted the fit slope from the monotonic numerosity response models for each adaptation. Within each visual field maps, we then select recording sites that meet the following criteria for further analysis: (1) where the preferred visual field position’s eccentricity is below 1°, i.e., recording sites whose visual spatial population receptive field include the numerosity stimulus area; (2) the slope of the monotonic model in the control condition is positive, so response amplitudes increase with numerosity; and (3) the model variance explained in the changing numerosity condition is at least 0.1. We then calculated the average slope among the selected voxels in each visual field map in each hemisphere (i.e., in each visual field map example) for each adaptor condition. Where the average variance explained in a visual field map example was below 0.1 in both the low and high adaptor conditions (indicating no clear responses to these conditions), we excluded that visual field map example from further analysis.

In subsequent analyses, for each visual field map, we use the resulting slope in each visual field map example as an independent measure. In each visual field map and adaptor condition, we first tested whether the slopes had a median significantly above zero using a two-sided Wilcoxon signed rank test. We then tested for significant differences between these slopes using the two-sided Wilcoxon signed rank test, where the values for each hemisphere in one adaptor condition and paired with the values from the same visual field map example in the other adaptor conditions, i.e., we tested whether the difference in these visual field map examples’ slopes between these two adaptor conditions was significantly above zero. As we performed this comparison separately for each visual field map, we performed a false discovery rate (FDR) correction^81^ on the resulting probability estimates, taking all visual field maps into account.

We also ask whether the strength of the adaptation effect on the monotonic model slope differed between visual field maps. This is complicated by the fact that, within each adaptor condition, all slopes show clear differences between visual field maps, making it difficult to interpret any changes between adaptor conditions. We would expect a visual field map with a high slope or high variance explained to be able to decrease this slope more (in absolute values) with adaptation. We therefore calculated the change in slope between the low and high adaptor conditions, and divided this by the slope in the changing adaptor condition to give a proportion by which the slope changed that was comparable between visual field maps. Having calculated the proportion by which the slope decreased in each visual field map example, we performed a two-factor ANOVA (factors: visual field map and participant) to test whether the proportional decrease in slope differs between visual field maps. These are corrected for multiple comparisons by using Tukey’s honestly significant difference test^82^, which gives the probabilities of the differences between the population marginal means shown in Fig. 5f and Supplementary Tables 6 and 8.

Next, we investigated the relationships between neural adaptation effects across different brain regions. First, we calculated the Pearson correlation of the proportional decrease in response slope for every pair of visual field maps (e.g., V1 vs. V2 in the same hemisphere). Next, we explored if these monotonic response decreases correlated with the neural adaptation effects on numerosity-tuned responses previously described in this dataset^44^. We observed that the variance explained by numerosity-tuned responses was consistently lower in the high adaptor condition, likely indicating greater response suppression. For each numerosity map and visual field map in one hemisphere, we paired this difference in variance explained and the proportional decrease in slope, respectively. Then, we computed the Pearson correlation of these paired values across hemispheres.

Finally, we examined the relationship between monotonic response slope effects and tuned numerosity preferences. Since adaptation effects on preferences involve both attractive and repulsive changes^44^, we summarized the effect strength by taking the signed difference between log numerosity preferences in low and high adaptor conditions for each voxel. Within each numerosity map’s responsive voxels, we then calculated the slope of the linear relationship between this difference and the log numerosity preference in the changing adaptor condition. For each numerosity map and visual field map in one hemisphere, we paired this slope (of preferred numerosity change) and the proportional decrease in slope (of montonic response amplitudes), respectively. Then, we computed the Pearson correlation of these paired values across hemispheres. For all correlations, we verified normality of residuals using an Anderson-Darling test, finding no significant deviations.

### Reporting summary

Further information on research design is available in the Nature Portfolio Reporting Summary linked to this article.

## Supplementary information

Supplementary Figs.

Reporting Summary

## Data Availability

The data set described in the study was also used in a previous publication^44^. Ethical constraints prevent us from sharing the medical imaging data sets (MRI scans) generated in the current study with public repositories. The structure of the brain is unique to the individual participant, in theory allowing the participant to be identified from these images, which may also contain medically sensitive findings. This is an interpretation of the EU’s General Data Protection Regulation (GDPR) for medical images, including MRI data. These raw data sets are available from the corresponding author upon reasonable request within a month, depending on agreements not to share these data publicly. Model parameters underlying all statistical analyses are available at (10.6084/m9.figshare.28633292). Response data for all model fitting are available at (10.6084/m9.figshare.28633322).

## Code availability

The code that supports the findings of this study is available from the following repositories: vistasoft (https://github.com/vistalab/vistasoft); vistasoftAddOns (https://github.com/benharvey/vistasoftAddOns); custom code for novel analyses (https://github.com/zhangliangysccd/NumerosityAdaptation).
