# Supplementary material for: Numerosity adaptation suppresses early visual responses
Source: Commun Biol. 2025 Nov 24;8:1655. doi: 10.1038/s42003-025-09041-4 (PMC12644745; doi:10.1038/s42003-025-09041-4)
Supplement: Supplementary file 1 — Supplementary Figs. [file 42003_2025_9041_MOESM1_ESM.pdf]

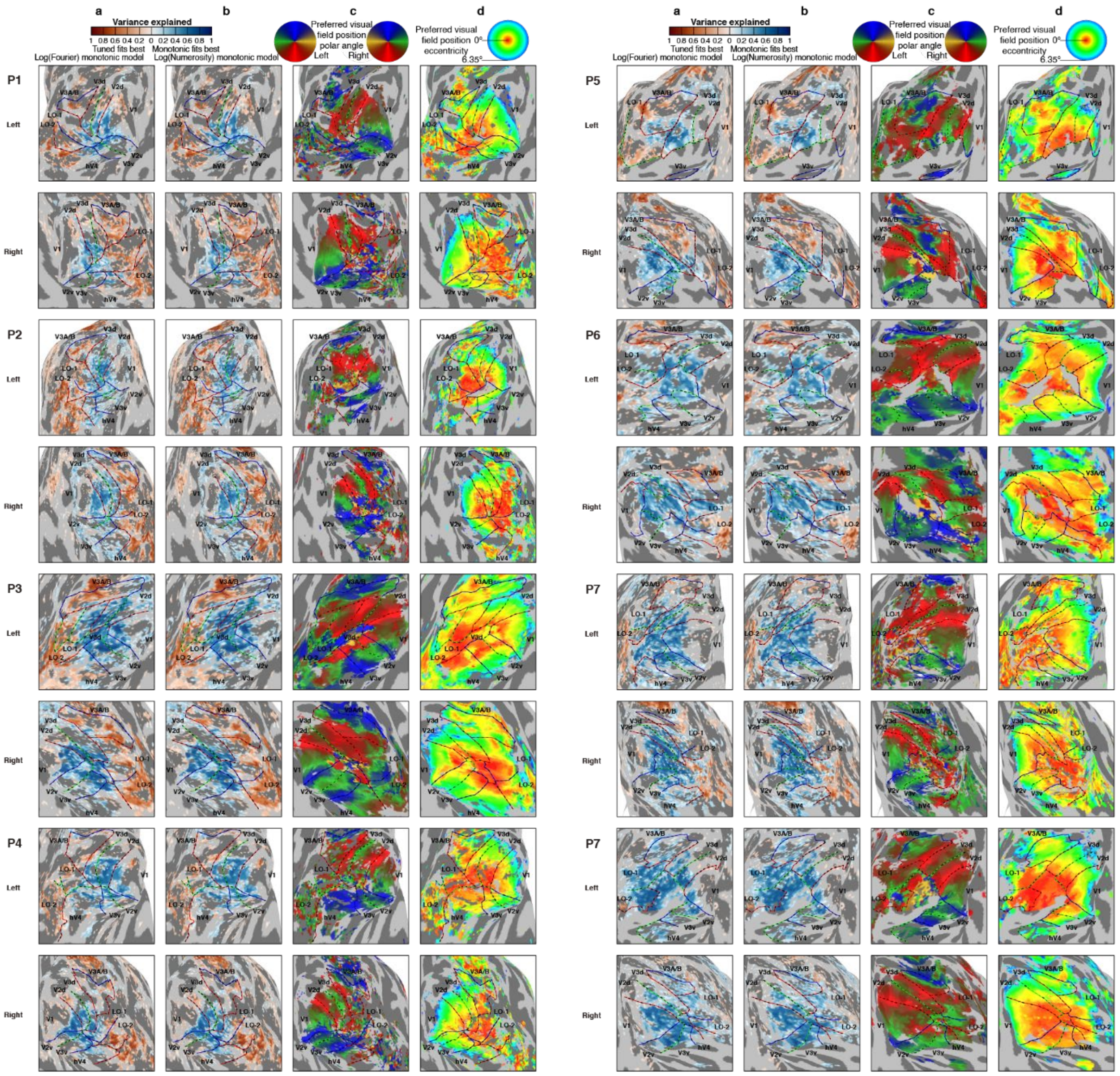

Supplementary Figure 1: Locations of monotonic responses to numerosity in all hemispheres. (**a**) Blue recording sites show responses that monotonically increased with numerosity (in proportion to the logarithm of aggregate Fourier power), while red recording sites show numerosity-tuned responses. Here, the best-fitting response model explained at least 0.1 (cross-validated R^2) of response variance. (**b**) Blue recording sites show responses that monotonically increased in proportion to the logarithm of numerosity, while red recording sites show numerosity-tuned responses. (**c**) The preferred visual field position polar angle of each recording site (obtained from visual field mapping data) let us localize visual field map borders at reversals in polar angle progressions. Dashed lines show visual field map borders at the upper vertical meridian (blue) lower vertical meridian (red) and horizontal meridian (green). (**d**) Each recording site's preferred visual field position eccentricity. We used this to localize sites with a preferred eccentricity below 1°, whose population receptive fields included the numerosity mapping stimulus area.

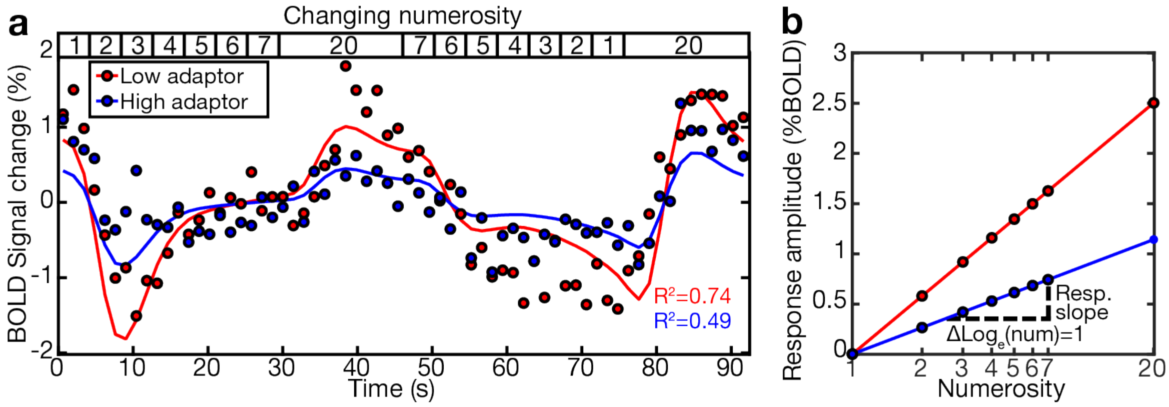

Supplementary Figure 2. The response of the V1 example recording site (voxel) used in Figure 4 to the numerosity mapping stimulus in each adaptor condition, with the predictions from a monotonic response proportional to log(numerosity). (**a**) As the stimulus' changing numerosity progressively increased and decreased (top inset), the fMRI BOLD response in all adaptation conditions (colored dots) increased and decreased, after a hemodynamic delay. The responses in all conditions were closely fit (R^2) by the predictions of the monotonic responses to log(numerosity) of the stimulus (colored lines), scaled with different amplitudes. However, these fits are slightly less good than the model following the logarithm of aggregate Fourier power (Figure 3). (**b**) As for the aggregate Fourier power model, we explained these responses using neural response models in which neural responses monotonically increase proportionally to the logarithm of the numerosity of the displays. We fit the slope of this proportionality (i.e. the increase in amplitude of the neural response when the logarithm of numerosity increases by one, ΔLog_e(num=1) using a general linear model. We can then compare these slopes between adaptor conditions.

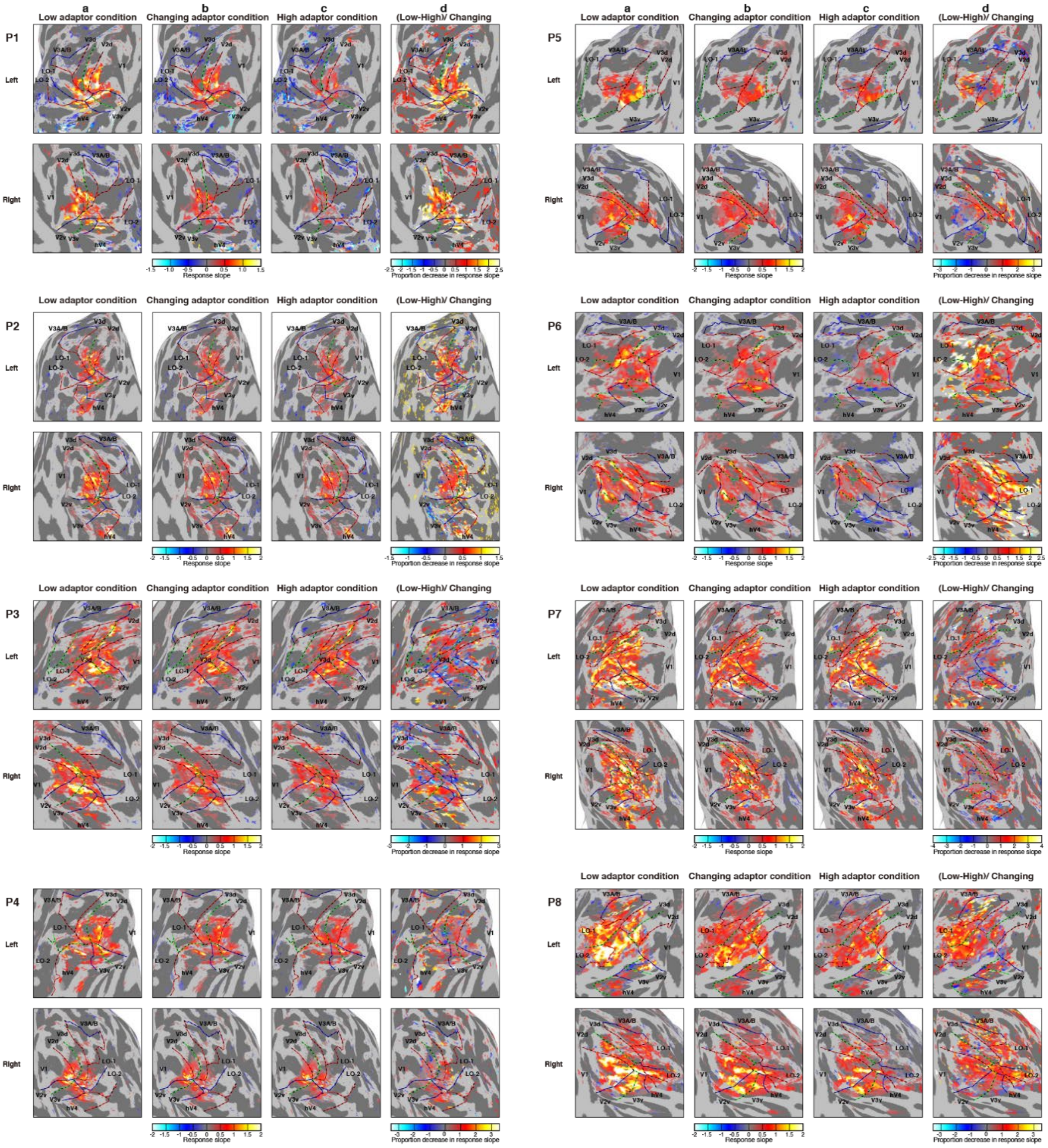

Supplementary Figure 3: Slope of proportionality between fMRI response amplitudes and the logarithm of aggregate Fourier power in every hemisphere and adaptor condition. (**a-c**) The fMRI BOLD responses increased monotonically with aggregate Fourier power in recording sites throughout the central visual field representations of the early visual field maps, in the low (a), changing (b) and high (c) adaptor conditions. The slope of this increase is greatest in the low adaptor condition, intermediate in the changing adaptor condition and smallest in the high adaptor condition (quantified in Figure 4d). (**d**) To compare this reduction in monotonic response amplitude between visual field maps, we calculated the change in response slope between adaptation conditions (here: low minus high) in each recording site, as a proportion of the slope in the changing adaptor condition. This proportional reduction in response amplitudes increased through the visual hierarchy (quantified in Figure 4f).

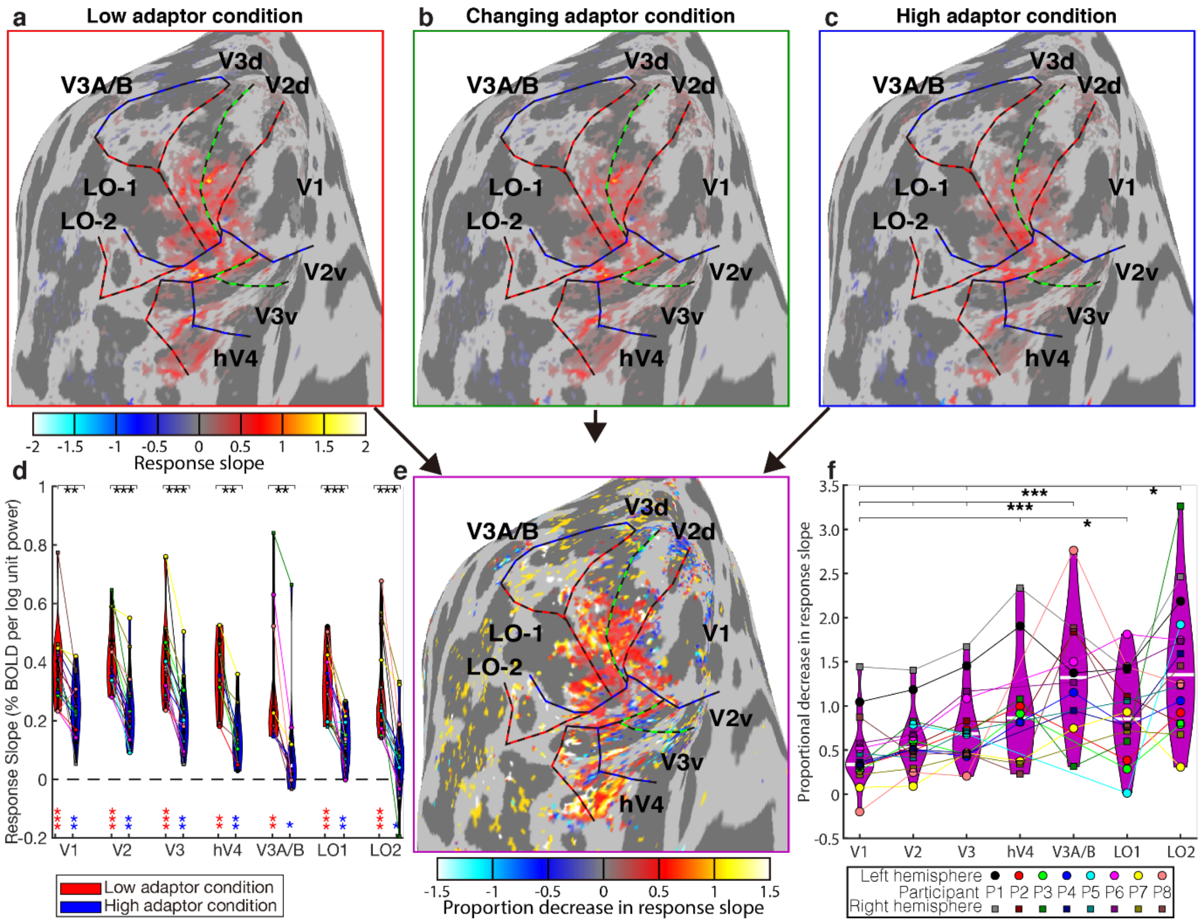

Supplementary Figure 4: Neural adaptation of monotonic responses increased through the visual hierarchy, in the monotonic model following the logarithm of numerosity. (**a-c**) The fMRI BOLD responses increased monotonically with numerosity in recording sites throughout the central visual field representations of the early visual field maps, in the low (a), changing (b) and high (c) adaptor conditions. (**d**) In the average across the recording sites in each visual field map of each hemisphere, the slope of the monotonic response increase with the logarithm of numerosity was significantly positive in all conditions and all visual field maps (colored stars, detailed statistics in Supplementary Table 1). This slope was greater in the low adaptor condition than the high adaptor condition (black stars show comparisons between conditions in each visual field map, detailed statistics in Supplementary Table 2) **p*<0.05, ***p*<0.01, ****p*<0.001. Colored markers (linked with colored lines) show the mean in the visual field map example in each hemisphere and condition. (**e**) As in the aggregate Fourier power model, we calculated the change in response slope between adaptation conditions (here: low minus high) in each recording site, as a proportion of the slope in the changing adaptor condition. (**f**) This proportional decrease in response amplitude from low to high adaptor conditions (i.e., the neural adaptation effect strength) became greater through the visual processing hierarchy. Visual field maps marked with brackets to the right of the stars showed significantly stronger proportional decreases than those with brackets to the left of the stars (detailed statistics in Supplementary Table 3-4).

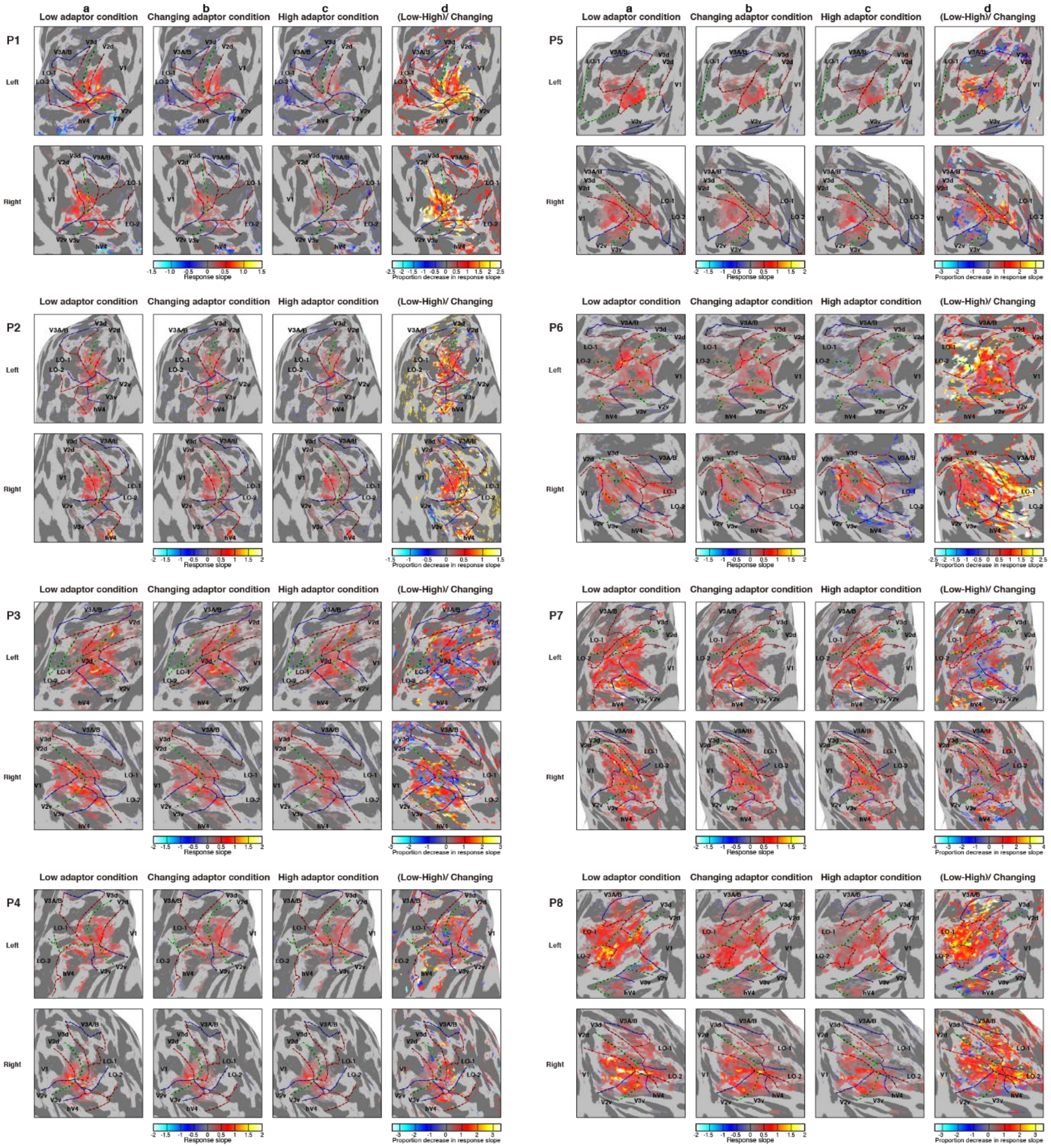

Supplementary Figure 5: Slope of proportionality between fMRI response amplitudes and the logarithm of numerosity in every hemisphere and adaptor condition. (**a-c**) As seen for the aggregate Fourier power model, the fMRI BOLD responses increased monotonically with numerosity in recording sites throughout the central visual field representations of the early visual field maps, in the low (a), changing (b) and high (c) adaptor conditions. The slope of this increase is greatest in the low adaptor condition, intermediate in the changing adaptor condition and smallest in the high adaptor condition (quantified in Supplementary Figure 4d). (**d**) To compare this reduction in monotonic response amplitude between visual field maps, we calculated the change in response slope between adaptation conditions (here: low minus high) in each recording site, as a proportion of the slope in the changing adaptor condition. This proportional reduction in response amplitudes increased through the visual hierarchy (quantified in Supplementary Figure 4f)

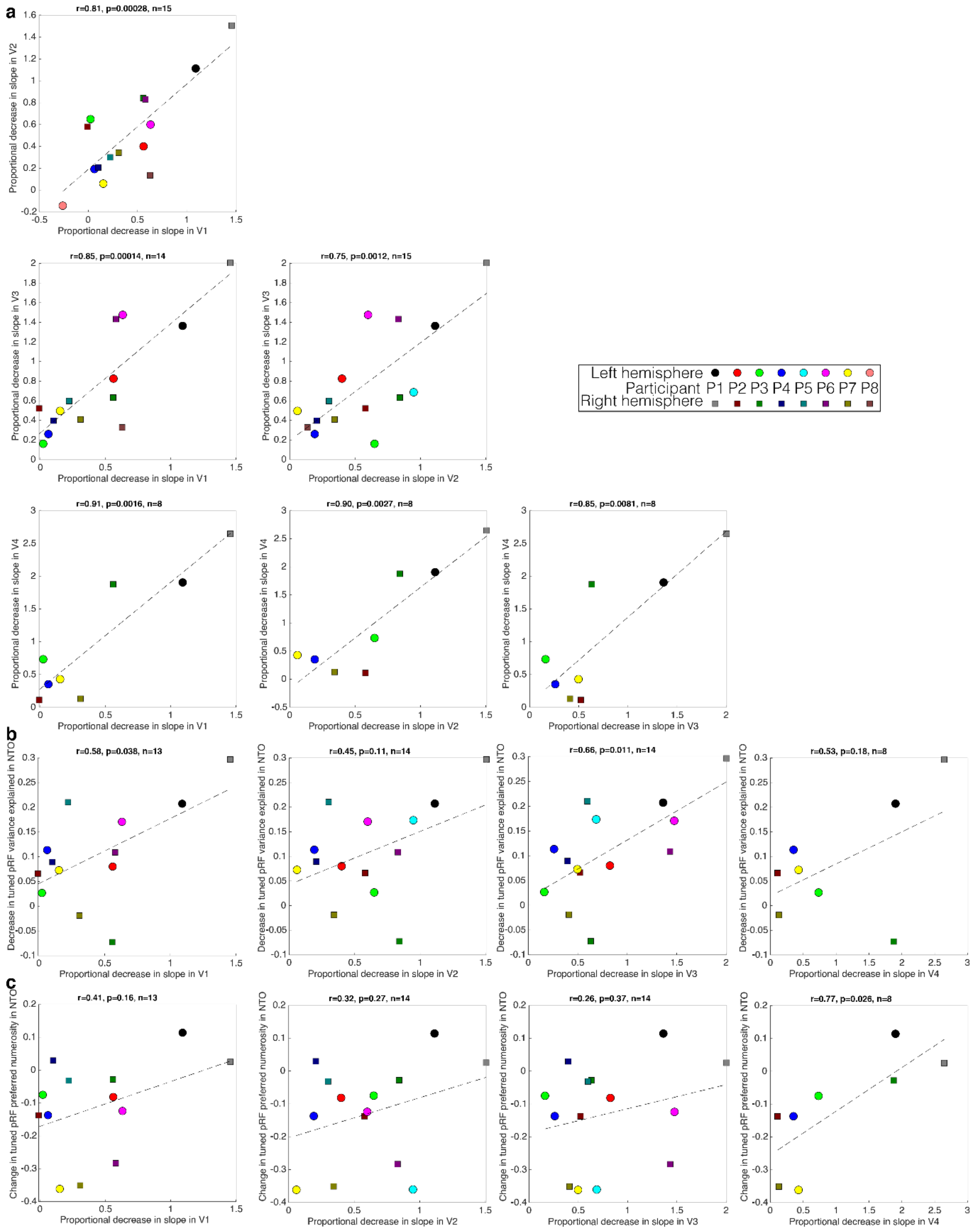

Supplementary Figure 6: Correlations between neural adaptation effect strengths in different brain areas, for the monotonic response model following aggregate Fourier power. (**a**) The proportional reduction in response amplitude was significantly correlated between V1, V2, V3 and hV4. Correlations between these early visual field maps and later visual field maps (LO1, LO2, V3A/B) and among these later visual field maps did not reach significance. (**b**) Adaptation to higher numerosities also reduced the variance explained by tuned response models in the topographic numerosity maps. Between hemispheres, the magnitude of this reduction in numerosity map NTO was significantly correlated with the proportional decrease in monotonic response slopes in V1 and V3 (but not V2 or hV4). The magnitude of this reduction in variance explained by tuned responses was not correlated between the different numerosity maps, and the reduction in other

numerosity maps was not correlated with the proportional decrease in monotonic response slopes in the early visual field maps. (**c**) Adaptation to higher numerosities also affect the numerosity preferences of voxels in the numerosity maps, with numerosity preferences attracted and repelled depending on the difference between the adaptor and the recording site's preferred numerosity (Tsouli et al., 2021). We summarized this effect by taking the slope of the difference in preferred numerosities in the low and high adaptor conditions, plotted against the preferred numerosity estimate from the changing numerosity adaptor condition. The magnitude of this change in numerosity preferences in NTO was significantly correlated with the proportional decrease in monotonic response slopes in hV4 only. The magnitude of this change in numerosity preferences was again not correlated between the different numerosity maps, and the reduction in other numerosity maps was not correlated with the proportional decrease in monotonic response slopes in the early visual field maps. Overall, these correlations are consistent with a relationship between neural adaptation effects at the monotonic and tuned stages (particularly in NTO), but our small sample of participants lacks the statistical power to confirm this link.

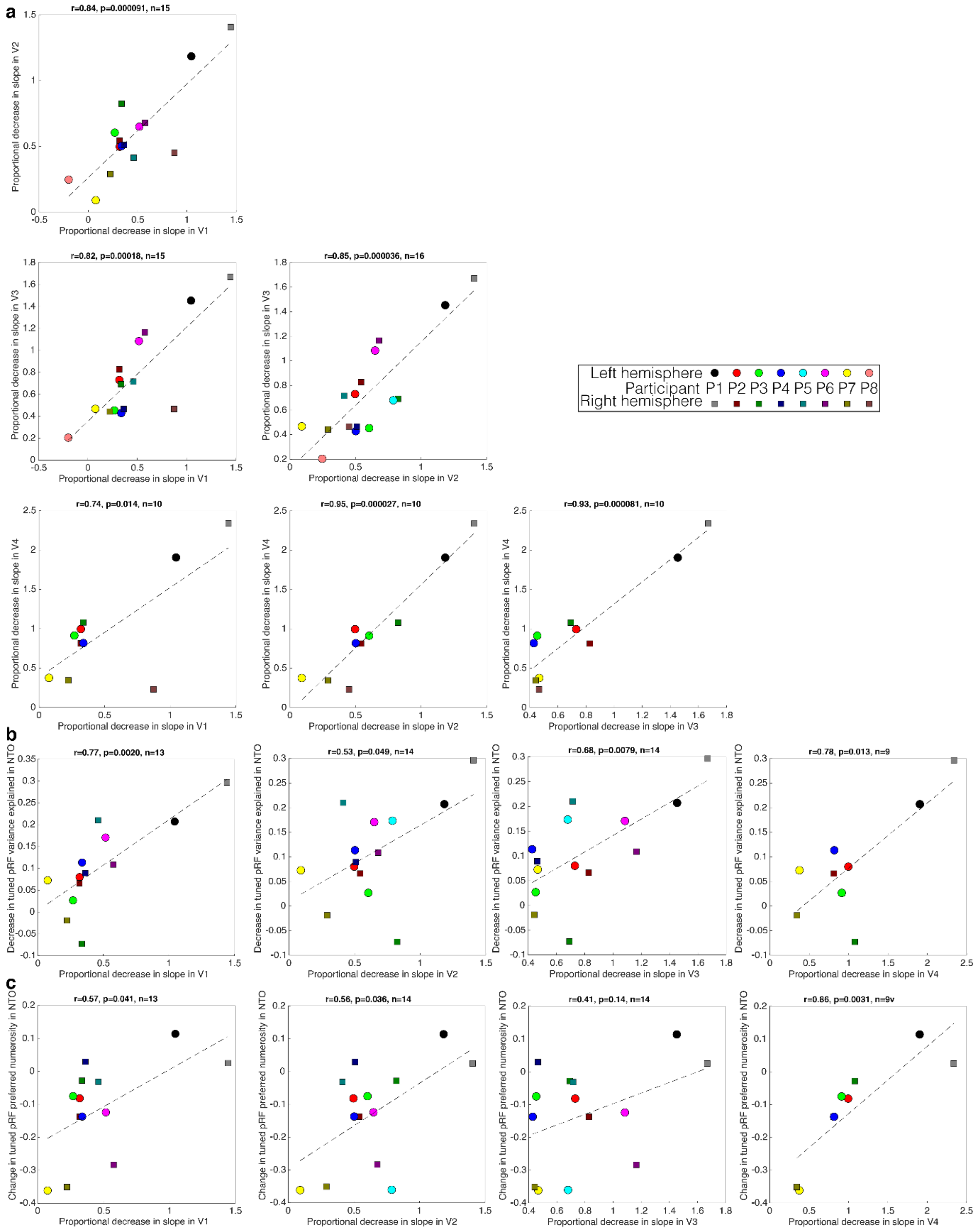

Supplementary Figure 7: Correlations between neural adaptation effect strengths in different brain areas, for the monotonic response model following numerosity. (**a**) The proportional reduction in response amplitude was significantly correlated between V1, V2, V3 and hV4. Correlations between these early visual field maps and later visual field maps (LO1, LO2, V3A/B) and among these later visual field maps did not reach significance. (**b**) Adaptation to higher numerosities also reduced the variance explained by tuned response models in the topographic numerosity maps. Between hemispheres, the magnitude of the reduction in variance explained between high and low adaptor condition in numerosity map NTO was significantly correlated with the proportional decrease in monotonic response slopes in V1, V2, V3 and hV4.

(**c**) The magnitude of the change in numerosity preferences in NTO was significantly correlated with the proportional decrease in monotonic response slopes in V1, V2 and hV4 (but not V3).

Supplementary Table 1: Descriptive and test statistics for the distribution of response slopes having a median above zero in each visual field map and adaptor condition, in the aggregate Fourier power model.

	Low adaptor				High adaptor			
Visual Field Maps	Median	*Z*	n	*p*	Median	*Z*	n	*p*
**V1**	0.825	3.41	15	0.0009	0.512	3.41	15	0.0011
**V2**	0.847	3.52	16	0.0008	0.470	3.52	16	0.0011
**V3**	0.845	3.52	16	0.0008	0.413	3.52	16	0.0011
**hV4**	0.748	2.80	10	0.0051	0.275	2.80	10	0.0071
**V3A/B**	0.482	2.93	11	0.0039	0.048	1.51	11	0.1307(ns)
**LO1**	0.714	3.52	16	0.0008	0.278	3.46	16	0.0011
**LO2**	0.532	3.52	16	0.0008	0.146	2.12	16	0.0397

Supplementary Table 2: Descriptive and test statistics for the distribution of differences between response slopes in each pair of conditions having a median above zero in each visual field map, in the aggregate Fourier power model.

	Low-High			
Visual Field Maps	Median	*Z*	n	*p*
**V1**	0.206	3.12	15	0.0025
**V2**	0.315	3.52	16	0.0008
**V3**	0.397	3.52	16	0.0008
**hV4**	0.378	2.80	10	0.0051
**V3A/B**	0.385	2.93	11	0.0039
**LO1**	0.362	3.52	16	0.0008
**LO2**	0.419	3.52	16	0.0008

Supplementary Table 3: Descriptive and test statistics for the distribution of response slopes having a median above zero in each visual field map and adaptor condition, in the log(numerosity).

	Low adaptor				High adaptor			
Visual Field Maps	Median	*Z*	n	*p*	Median	*Z*	n	*p*
**V1**	0.371	3.41	15	0.0009	0.229	3.41	15	0.0011
**V2**	0.377	3.52	16	0.0008	0.210	3.52	16	0.0011
**V3**	0.379	3.52	16	0.0008	0.186	3.52	16	0.0011
**hV4**	0.340	2.80	10	0.0051	0.125	2.80	10	0.0071
**V3A/B**	0.225	2.80	10	0.0051	0.042	1.99	10	0.0469
**LO1**	0.318	3.52	16	0.0008	0.123	3.46	16	0.0011
**LO2**	0.238	3.52	16	0.0008	0.070	2.07	16	0.0450

Supplementary Table 4: Descriptive and test statistics for the distribution of differences between response slopes in each pair of conditions having a median above zero in each visual field map, in the log(numerosity).

	Low-High			
Visual Field Maps	Median	*Z*	n	*p*
**V1**	0.091	3.12	15	0.0025
**V2**	0.141	3.52	16	0.0008
**V3**	0.178	3.52	16	0.0008
**hV4**	0.168	2.80	10	0.0051
**V3A/B**	0.196	2.80	10	0.0051
**LO1**	0.163	3.52	16	0.0008
**LO2**	0.184	3.52	16	0.0008

Supplementary Table 5: Pairwise mean differences between the proportional decrease in response slopes in different visual field maps, in the aggregate Fourier power model.

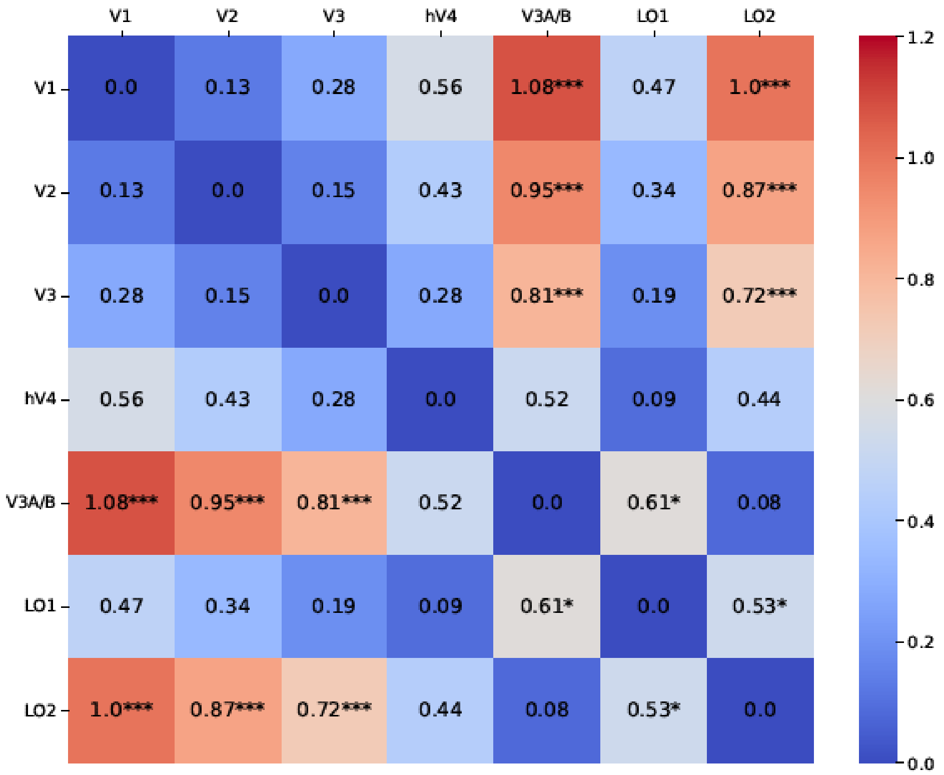

Visual Field Maps	V1	V2	V3	hV4	V3A/B	LO1	LO2
**V1**	0	-0.13	-0.28	-0.56	-1.08	-0.47	-1.00
**V2**	0.13	0	-0.15	-0.43	-0.95	-0.34	-0.87
**V3**	0.28	0.15	0	-0.28	-0.81	-0.19	-0.72
**hV4**	0.56	0.43	0.28	0	-0.52	0.09	-0.44
**V3A/B**	1.08	0.95	0.81	0.52	0	0.61	0.08
**LO1**	0.47	0.34	0.19	-0.09	-0.61	0	-0.53
**LO2**	1.00	0.87	0.72	0.44	-0.08	0.53	0

Supplementary Table 6: Statistical significance of differences between the proportional decrease in response slopes in different visual field maps in Tukey's honestly significant difference test, in the aggregate Fourier power model.

Visual Field Maps	V1	V2	V3	hV4	V3A/B	LO1	LO2
**V1**	1.000000	0.985248	0.638428	0.061994	0.000001	0.082390	0.000001
**V2**	0.985248	1.000000	0.972671	0.272569	0.000022	0.380277	0.000015
**V3**	0.638428	0.972671	1.000000	0.747534	0.000500	0.901349	0.000485
**hV4**	0.061994	0.272569	0.747534	1.000000	0.155799	0.999065	0.230114
**V3A/B**	0.000001	0.000022	0.000500	0.155799	1.000000	0.017519	0.999402
**LO1**	0.082390	0.380277	0.901349	0.999065	0.017519	1.000000	0.024242
**LO2**	0.000001	0.000015	0.000485	0.230114	0.999402	0.024242	1.000000

Supplementary Table 7: Pairwise mean differences between the proportional decrease in response slopes in different visual field maps, in the log(numerosity).

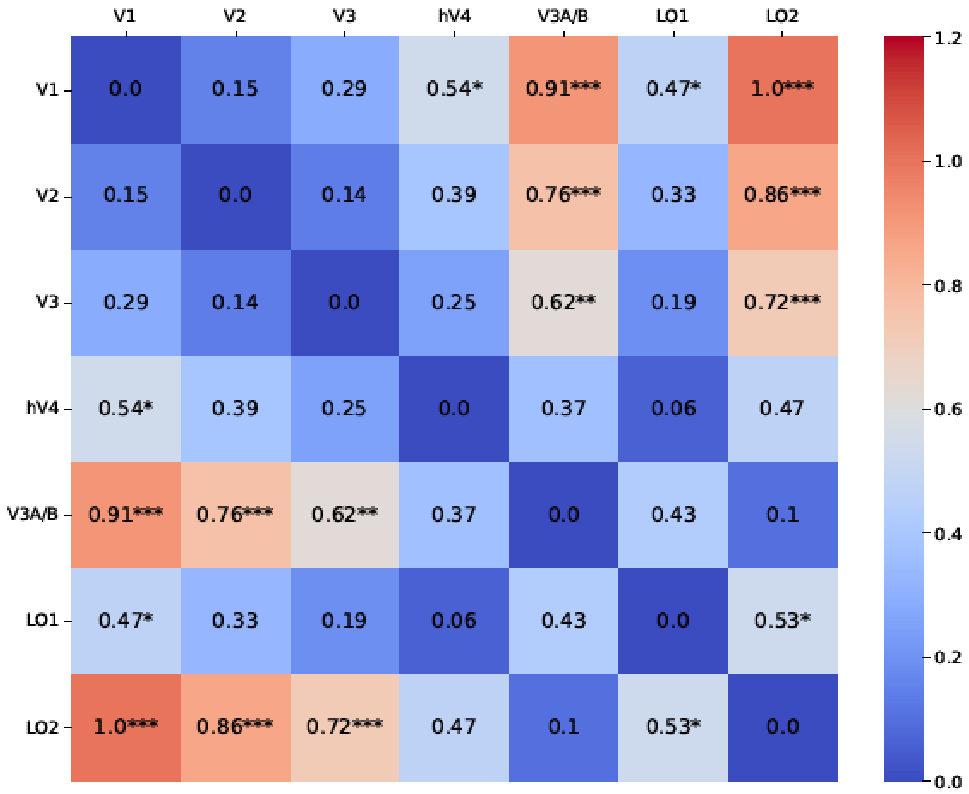

Visual Field Maps	V1	V2	V3	hV4	V3A/B	LO1	LO2
**V1**	0	-0.15	-0.29	-0.54	-0.91	-0.47	-1.00
**V2**	0.15	0	-0.14	-0.39	-0.76	-0.33	-0.86
**V3**	0.29	0.14	0	-0.25	-0.62	-0.19	-0.72
**hV4**	0.54	0.39	0.25	0	-0.37	0.06	-0.47
**V3A/B**	0.91	0.76	0.62	0.37	0	0.43	-0.10
**LO1**	0.47	0.33	0.19	-0.06	-0.43	0	-0.53
**LO2**	1.00	0.86	0.72	0.47	0.10	0.53	0

Supplementary Table 8: Statistical significance of differences between the proportional decrease in response slopes in different visual field maps in Tukey's honestly significant difference test, in the log(numerosity).

Visual Field Maps	V1	V2	V3	hV4	V3A/B	LO1	LO2
**V1**	1.00000000	0.96212985	0.49830682	0.04514129	0.00002663	0.04046189	0.00000008
**V2**	0.96212985	1.00000000	0.96420820	0.28364971	0.00058891	0.31538371	0.00000330
**V3**	0.49830682	0.96420820	1.00000000	0.78451180	0.00965879	0.87638528	0.00015112
**hV4**	0.04514129	0.28364971	0.78451180	1.00000000	0.47364830	0.99982266	0.11384435
**V3A/B**	0.00002663	0.00058891	0.00965879	0.47364830	1.00000000	0.16858939	0.99790149
**LO1**	0.04046189	0.31538371	0.87638528	0.99982266	0.16858939	1.00000000	0.01162116
**LO2**	0.00000008	0.00000330	0.00015112	0.11384435	0.99790149	0.01162116	1.00000000
