# Supplementary material for: Numerosity adaptation suppresses early visual responses
Source: Commun Biol. 2025 Nov 24;8:1655. doi: 10.1038/s42003-025-09041-4 (PMC12644745; doi:10.1038/s42003-025-09041-4)
Supplement: Supplementary file 2 — Reporting Summary [file 42003_2025_9041_MOESM2_ESM.pdf]

nature portfolio

Corresponding author(s): Liangyou Zhang

Last updated by author(s): Aug 27, 2025

Reporting Summary

Nature Portfolio wishes to improve the reproducibility of the work that we publish. This form provides structure for consistency and transparency in reporting. For further information on Nature Portfolio policies, see our Editorial Policies and the Editorial Policy Checklist.

Statistics

For all statistical analyses, confirm that the following items are present in the figure legend, table legend, main text, or Methods section.

n/a	Confirmed	
☐	☒	The exact sample size (*n*) for each experimental group/condition, given as a discrete number and unit of measurement
☐	☒	A statement on whether measurements were taken from distinct samples or whether the same sample was measured repeatedly
☐	☒	The statistical test(s) used AND whether they are one- or two-sided<br>*Only common tests should be described solely by name; describe more complex techniques in the Methods section.*
☒	☐	A description of all covariates tested
☐	☒	A description of any assumptions or corrections, such as tests of normality and adjustment for multiple comparisons
☐	☒	A full description of the statistical parameters including central tendency (e.g. means) or other basic estimates (e.g. regression coefficient) AND variation (e.g. standard deviation) or associated estimates of uncertainty (e.g. confidence intervals)
☐	☒	For null hypothesis testing, the test statistic (e.g. F, t, r) with confidence intervals, effect sizes, degrees of freedom and P value noted<br>*Give P values as exact values whenever suitable.*
☒	☐	For Bayesian analysis, information on the choice of priors and Markov chain Monte Carlo settings
☒	☐	For hierarchical and complex designs, identification of the appropriate level for tests and full reporting of outcomes
☒	☐	Estimates of effect sizes (e.g. Cohen's *d*, Pearson's *r*), indicating how they were calculated

Our web collection on statistics for biologists contains articles on many of the points above.

Software and code

Policy information about availability of computer code

Data collection	7T Philips Achieva scanner, PsychToolbox-3, in-house IDL software (v6.3, RSI, Boulder, CO, USA)
Data analysis	For initial data (pre-)processing:<br>mrVista 2.0 (https://github.com/vistalab/vistasoft)<br>Matlab version 2022a (The Mathworks)<br>Freesurfer 6.0 (https://surfer.nmr.mgh.harvard.edu/)<br>ITK-SNAP 1.6.0.1<br>AFNI 19.1.21 (afni.nimh.nih.gov)<br><br>For later data analyses:<br>Matlab version 2022a (The Mathworks)<br><br>The code that supports the findings of this study is available from the following repositories: vistasoft (https://github.com/vistalab/vistasoft); vistasoftAddOns (https://github.com/benharvey/vistasoftAddOns); custom code for novel analyses (https://github.com/zhangliangysccd/NumerosityAdaptation).

For manuscripts utilizing custom algorithms or software that are central to the research but not yet described in published literature, software must be made available to editors and reviewers. We strongly encourage code deposition in a community repository (e.g. GitHub). See the Nature Portfolio guidelines for submitting code & software for further information.

Data

Policy information about availability of data

All manuscripts must include a data availability statement. This statement should provide the following information, where applicable:

- Accession codes, unique identifiers, or web links for publicly available datasets
- A description of any restrictions on data availability
- For clinical datasets or third party data, please ensure that the statement adheres to our policy

Ethical constraints prevent us from sharing the medical imaging data sets (MRI scans) generated in the current study to public repositories. The structure of the brain is unique to the individual participant, in theory allowing the participant to be identified from these images, which may also contain medically sensitive findings. This is an interpretation of the EU's General Data Protection Regulation (GDPR) for medical images including MRI data. These raw data sets are available from the corresponding author upon reasonable request within a month, depending on agreements not to share these data publicly. Model parameters underlying The data set described in the study was also used in a previous publication. Ethical constraints prevent us from sharing the medical imaging data sets (MRI scans) generated in the current study to public repositories. The structure of the brain is unique to the individual participant, in theory allowing the participant to be identified from these images, which may also contain medically sensitive findings. This is an interpretation of the EU's General Data Protection Regulation (GDPR) for medical images including MRI data. These raw data sets are available from the corresponding author upon reasonable request within a month, depending on agreements not to share these data publicly. Model parameters underlying all statistical analyses are available at (10.6084/m9.figshare.28633292). Response data for all model fitting are available at (10.6084/m9.figshare.28633322).

Research involving human participants, their data, or biological material

Policy information about studies with human participants or human data. See also policy information about sex, gender (identity/presentation), and sexual orientation and race, ethnicity and racism.

Reporting on sex and gender	No sex or gender related analyses were performed. Our sample includes similar numbers of male and female participants (five male, three female).
Reporting on race, ethnicity, or other socially relevant groupings	None
Population characteristics	None
Recruitment	Participants were recruited by volunteering, from a population of local students and researchers (a convenience sample). All participants were right-handed and had normal or corrected to normal vision. The rationale for selecting a research sample of healthy neurotypical participants is that we were interested in studying normal brain function. As we do not expect differences in the representations of timing in the brain between sexes, age, or educational level, we consider this sample representative.
Ethics oversight	All experimental procedures were approved by the ethics committee of University Medical Center Utrecht (protocol number 09/350).

Note that full information on the approval of the study protocol must also be provided in the manuscript.

Field-specific reporting

Please select the one below that is the best fit for your research. If you are not sure, read the appropriate sections before making your selection.

☐ Life sciences ☒ Behavioural & social sciences ☐ Ecological, evolutionary & environmental sciences

For a reference copy of the document with all sections, see nature.com/documents/nr-reporting-summary-flat.pdf

Behavioural & social sciences study design

All studies must disclose on these points even when the disclosure is negative.

Study description	Here we ask whether and how monotonic and tuned neural responses to visual numerosity are affected by numerosity adaptation, throughout the brain's hierarchy of both visual field maps and numerosity maps. We answer these questions by reanalyzing ultra-high-field (7T) functional magnetic resonance imaging (fMRI) data that was acquired during the presentation of visual displays that gradually varied in numerosity, alternative with different adapter numerosities. We compare the response amplitudes of monotonically increasing neural response models throughout the early visual system to reveal how these responses are affected by the adaptor's numerosity, and how these effects change between visual field maps.
Research sample	We acquired fMRI data from eight participants (3 female, aged 26 to 52). All participants were right-handed and had normal or corrected to normal vision. The rationale for selecting a research sample of healthy neurotypical participants is that we were interested in studying normal brain function. As we do not expect differences in the representations of numerosity in the brain between sexes, age, or educational level, we consider this sample representative.

Sampling strategy	We used a convenience sample of 8 researchers. Two participants were co-authors, familiar with the goals of the study. The remaining six were graduate students from elsewhere in our university and hospital recruited through advertising. No sample size calculation was performed, as all data was originally collected for another study (Touli et al, 2021, NeuroImage). In this other study, sample sizes were chosen based on the sample sizes that had previously been used to convincingly demonstrate reproducibility in experiments with similar designs throughout the field.
Data collection	We acquired MRI data on a 7T Philips Achieva scanner.<br><br>No one else was present besides the participant(s) and the researchers during data collection. This data was originally collected for another study, where the operator was aware of the hypothesis of that study. This hypothesis entailed the existence of adaptation effects on timing-selective response. The researchers were not blinded to the experimental conditions being compared because the researcher's knowledge of these conditions could not affect the amplitude of the participants' brain responses.
Timing	Data were collected from 30/06/2019 to 14/04/2020.
Data exclusions	Data from all scanned participants was included. However, we acquired data from the whole brain, although only a small set of regions was analyzed, as in all fMRI experiments. Most locations in the brain do not respond to the changes in numerosity. We first excluded from analysis any recording sites (voxels) that lay outside the gray matter. The models were fit on all included, gray matter voxels. For further analyses where model fits were compared (except the surface renderings), voxels outside of visual field maps or numerosity maps were excluded. For all model comparisons, we also excluded voxels where the variance explained the response models was 0.1 or less. This was in total several million excluded voxels.
Non-participation	No participants dropped out/declined participation
Randomization	Participants were not allocated into experimental groups

Reporting for specific materials, systems and methods

We require information from authors about some types of materials, experimental systems and methods used in many studies. Here, indicate whether each material, system or method listed is relevant to your study. If you are not sure if a list item applies to your research, read the appropriate section before selecting a response.

Materials & experimental systems

n/a	Involved in the study
☒	☐ Antibodies
☒	☐ Eukaryotic cell lines
☒	☐ Palaeontology and archaeology
☒	☐ Animals and other organisms
☒	☐ Clinical data
☒	☐ Dual use research of concern
☒	☐ Plants

Methods

n/a	Involved in the study
☒	☐ ChIP-seq
☒	☐ Flow cytometry
☐	☒ MRI-based neuroimaging

Plants

Seed stocks	*Report on the source of all seed stocks or other plant material used. If applicable, state the seed stock centre and catalogue number. If plant specimens were collected from the field, describe the collection location, date and sampling procedures.*
Novel plant genotypes	*Describe the methods by which all novel plant genotypes were produced. This includes those generated by transgenic approaches, gene editing, chemical/radiation-based mutagenesis and hybridization. For transgenic lines, describe the transformation method, the number of independent lines analyzed and the generation upon which experiments were performed. For gene-edited lines, describe the editor used, the endogenous sequence targeted for editing, the targeting guide RNA sequence (if applicable) and how the editor was applied.*
Authentication	*Describe any authentication procedures for each seed stock used or novel genotype generated. Describe any experiments used to assess the effect of a mutation and, where applicable, how potential secondary effects (e.g. second site T-DNA insertions, mosiacism, off-target gene editing) were examined.*

Magnetic resonance imaging

Experimental design

Design type	Task fMRI
Design specifications	During fMRI scanning, participants viewed sequences of progressively increasing and decreasing numerosities (from one to seven and back) to quantify response amplitudes to different numerosities. These progressively changing numerosity displays were presented in three different adaptor conditions (Fig. 1): (1) Preceded by displays containing one item (low

adaptor condition); (2) Preceded by displays containing twenty items (high adaptor condition); (3) Preceded by displays of the same changing numerosity (changing adaptor condition).
In each scan session, we also acquired a top-up scan with the opposite phase-encoding direction to correct for image distortion in the gradient encoding direction, and a T1-weighted anatomical image with the same resolution, position and orientation as the functional data.
For visual field mapping, a bar filled with a moving checkerboard pattern stepped across a 6.35° (radius) circle in the display center in eight (cardinal and diagonal) directions.

Behavioral performance measures: During visual field mapping the central fixation cross changed color (red/green) and during presentation of numerosity stimuli the dots changed color (from black to white) at pseudo-random intervals (on average once every 21 seconds). Participants were instructed to press a button when a color change occurred to confirm they were paying attention to the stimuli and remained awake throughout scanning.

Acquisition

Imaging type(s): Functional

Field strength: 7 Tesla

Sequence & imaging parameters: Functional T2*-weighted 2D echo planar images were acquired using multiband acquisition (multiband factor: 2) and anterior-posterior encoding, and a 32-channel head coil, at a resolution of 1.77 × 1.77 × 1.75 mm, with a field of view of 227 × 227 × 70 mm. The TR was 1400 ms, echo time (TE) was 25 ms, and flip angle was 70°. Functional runs were each 273 time frames (382.2 s) in duration, of which the first 9 time frames (12.6 s) were discarded to ensure the signal was at steady state.

Area of acquisition: Whole brain

Diffusion MRI: ☐ Used ☒ Not used

Preprocessing

Preprocessing software: Freesurfer 6.0 (https://surfer.nmr.mgh.harvard.edu/), ITK-SNAP 1.6.0.1, AFNI 19.1.21 (afni.nimh.nih.gov)

Normalization: Analyses were performed in each participants' native space

Normalization template: Analyses were performed in each participants' native space

Noise and artifact removal: Functional scans were corrected for head movement and motion with two series of images that were acquired using opposing phase-encoding directions, with transformations calculated using AFNI (3dvolreg, 3dQwarp, 3dNwarpApply). No other spatial or temporal smoothing procedures were applied

Volume censoring: There was no volume censoring

Statistical modeling & inference

Model type and settings: Population receptive field modeling and general linear models

Effect(s) tested: Comparison of model response amplitudes (beta parameters in general linear models).

Specify type of analysis: ☐ Whole brain ☒ ROI-based ☐ Both

Anatomical location(s): Visual field maps

Statistic type for inference: Visual field map-wise

(See Eklund et al. 2016)

Correction: The voxel-wise population receptive field model and monotonic response model fits were not corrected for multiple comparisons, but all following statistics on visual field maps and numerosity maps were

Models & analysis

n/a	Involved in the study
☒	☐ Functional and/or effective connectivity
☒	☐ Graph analysis
☐	☒ Multivariate modeling or predictive analysis

Multivariate modeling and predictive analysis: Population receptive field modeling and general linear models
